# *Pf*ATP4 inhibitors in the Medicines for Malaria Venture Malaria Box and Pathogen Box block the schizont-to-ring transition by inhibiting egress rather than invasion

**DOI:** 10.3389/fcimb.2022.1060202

**Published:** 2022-11-30

**Authors:** Claudia B. G. Barnes, Madeline G. Dans, Thorey K. Jonsdottir, Brendan S. Crabb, Paul R. Gilson

**Affiliations:** ^1^ Life Sciences, Burnet Institute, Melbourne, VIC, Australia; ^2^ Department of Medicine, The University of Melbourne, Melbourne, VIC, Australia; ^3^ School of Medicine, Deakin University, Geelong, VIC, Australia; ^4^ Department of Microbiology and Immunology, The University of Melbourne, Parkville, VIC, Australia; ^5^ Department of Immunology and Pathology, Monash University, Melbourne, VIC, Australia

**Keywords:** *Plasmodium falciparum*, malaria, *Pf*ATP4, sodium ions, egress, invasion, Malaria Box, Pathogen Box

## Abstract

The cation efflux pump *Plasmodium falciparum* ATPase 4 (*Pf*ATP4) maintains Na^+^ homeostasis in malaria parasites and has been implicated in the mechanism of action of many structurally diverse antimalarial agents, including >7% of the antimalarial compounds in the Medicines for Malaria Venture’s ‘Malaria Box’ and ‘Pathogen Box’. Recent screens of the ‘Malaria Box’ and ‘Pathogen Box’ revealed that many *Pf*ATP4 inhibitors prevent parasites from exiting their host red blood cell (egress) or entering new host cells (invasion), suggesting that these compounds may have additional molecular targets involved in egress or invasion. Here, we demonstrate that five *Pf*ATP4 inhibitors reduce egress but not invasion. These compounds appear to inhibit egress by blocking the activation of protein kinase G, an enzyme that, once stimulated, rapidly activates parasite egress. We establish a direct link between egress and *Pf*ATP4 function by showing that the inhibition of egress is attenuated in a Na^+^-depleted environment and in parasites with a mutation in *pfatp4*. Finally, we show that *Pf*ATP4 inhibitors induce host cell lysis when administered prior to the completion of parasite replication. Since host cell lysis mimics egress but is not followed by invasion, this phenomenon likely explains why several *Pf*ATP4 inhibitors were previously classified as invasion inhibitors. Collectively, our results confirm that *Pf*ATP4-mediated Na^+^ efflux is critical to the regulation of parasite egress.

## 1 Introduction

In 2020, there were 241 million cases of malaria, a life-threatening disease caused by Apicomplexan parasites of the genus *Plasmodium* (World Health Organization, 2022). Although the global burden of malaria has fallen dramatically since the beginning of this century, largely due to vector control strategies and the widespread availability of artemisinin-based combination therapies (ACTs) (Roser and Ritchie, 2015; World Health Organization, 2021), ongoing progress towards eradication is threatened by the spread of ACT-resistant parasites in Asia and Africa (Amato et al., 2018; Balikagala et al., 2021). The 12% increase in malaria-attributable deaths in 2020 compared to 2019 is a timely reminder of the urgent need to develop antimalarial drugs with novel mechanisms of action (MoA), particularly to target the most virulent malaria parasite, *P. falciparum* (Sato, 2021; World Health Organization, 2021).

Between 2008 and 2011, almost 6 million compounds were tested for activity against blood-stage *P. falciparum* in whole-cell phenotypic screens, yielding some 20 000 unique hits (Gamo et al., 2010; Guiguemde et al., 2010; Meister et al., 2011). To render these compounds accessible to researchers without the capacity to study vast compound libraries, the organisation Medicines for Malaria Venture (MMV) compiled the Malaria Box, a collection of 400 hit antimalarials with unknown MoA, prioritised based on their structural diversity and *in vitro* potency against asexual intraerythrocytic *P. falciparum* (Spangenberg et al., 2013). A second library, the Pathogen Box, was subsequently released, containing compounds active against malaria and other pathogens (Medicines for Malaria Venture, 2022). With a view to identifying probes for studying essential parasite processes as well as candidates for medicinal chemistry optimisation and eventual drug development, these libraries have been screened for activity against specific life-cycle stages (Lucantoni et al., 2013; Van Voorhis et al., 2016; Patra et al., 2020; Kanatani et al., 2022) and parasite activities (Fong et al., 2015; Ahyong et al., 2016; Looker et al., 2022).

Both the Malaria Box and the Pathogen Box have been screened for compounds that exert their parasiticidal effects by inhibiting the active cation transporter *P. falciparum* ATPase 4 (*Pf*ATP4), a protein which has been implicated in the MoA of structurally diverse clinical (Rottmann et al., 2010; Jiménez-Díaz et al., 2014) and preclinical antimalarials (Vaidya et al., 2014; Flannery et al., 2015; Gilson et al., 2019). *Pf*ATP4 localises to the parasite plasma membrane and extrudes Na^+^ from the parasite in exchange for H^+^, thereby maintaining an intra-parasitic Na^+^ concentration approximately ten times lower than that of the host cell’s cytoplasm while imposing an ‘acid load’ on the parasite (Spillman et al., 2013a; Spillman et al., 2013b). *Pf*ATP4 inhibitors trigger a rapid increase in the intra-parasitic Na^+^ concentration and pH (Spillman et al., 2013b; Jiménez-Díaz et al., 2014; Lehane et al., 2014; Flannery et al., 2015) and cause swelling (Jiménez-Díaz et al., 2014; Dennis et al., 2018a; Dennis et al., 2018b), sometimes resulting in lysis (Jiménez-Díaz et al., 2014; Gilson et al., 2019), of isolated parasites and infected red blood cells (RBCs), likely due to the osmotic influx of water that accompanies Na^+^ elevation. Based on their induction of this distinctive biochemical signature, 7% and 9% of the antimalarials in the Malaria Box and Pathogen Box, respectively, were classified as “*Pf*ATP4-associated compounds” (Lehane et al., 2014; Dennis et al., 2018b). The frequency with which structurally diverse experimental antimalarials seem to target *Pf*ATP4 testifies to the importance of *Pf*ATP4-regulated Na^+^ homeostasis for parasite viability and to the unique druggability of *Pf*ATP4.

While the effects of Na^+^-perturbing compounds on intraerythrocytic *P. falciparum* are well studied, recent evidence suggests that these compounds also disrupt the brief window in the parasite’s blood-stage cell cycle during which merozoites are released from their schizonts and rapidly invade new host RBCs. There is an incentive to discover novel compounds that act against blood-stage merozoite egress and invasion as these compounds could synergise with current antimalarials that primarily target intraerythrocytic parasite development (reviewed by (Tse et al., 2019)). Furthermore, these precisely orchestrated pathways present multiple potential drug targets (Burns et al., 2019; Dvorin and Goldberg, 2022), some of which are conserved across parasite isolates and species (Baum et al., 2006; Liffner et al., 2020; Bahl et al., 2021). In 2018, Subramanian et al. (2018) screened the Malaria Box for inhibitors of the schizont-to-ring transition and found that ten of the twenty-eight putative *Pf*ATP4 inhibitors (Lehane et al., 2014) suppressed egress or invasion. A comparable screen of the Pathogen Box (Dans et al., 2020) revealed that out of eleven *Pf*ATP4 inhibitors (Dennis et al., 2018b), six reduced egress and four reduced invasion. While these results provide compelling evidence for a link between *Pf*ATP4 and egress/invasion, the mechanistic basis for this link remains unresolved. Moreover, the observation that some *Pf*ATP4 inhibitors reduced egress while others targeted invasion suggested that the effects of *Pf*ATP4 inhibitors during the schizont-to-ring transition may be attributable to targets additional to *Pf*ATP4 which either modulate the effects of Na^+^ dyshomeostasis on egress and invasion or inhibit egress and invasion independently of *Pf*ATP4.

In this study, five putative *Pf*ATP4 inhibitors from the Malaria and Pathogen Boxes (Lehane et al., 2014; Dennis et al., 2018b) (**Supplementary Figure 1**) with previously published effects on egress and invasion (Subramanian et al., 2018; Dans et al., 2020) were selected based on structural diversity and commercial availability and administered to parasites at specific points during the schizont-to-ring transition. All five compounds were found to arrest egress and not RBC invasion or early ring-stage development. We provide evidence to suggest that *Pf*ATP4 inhibitors block egress by preventing the activation of cyclic guanosine monophosphate (cGMP)-dependent protein kinase G (*Pf*PKG), an essential enzyme that is activated minutes before merozoites are released (Collins et al., 2013; Koussis et al., 2020; Dvorin and Goldberg, 2022). Further investigation revealed a reduction in the potency of egress inhibition in a Na^+^-depleted growth medium and in parasites with a resistance-conferring mutation in *Pf*ATP4, indicating that inhibition of *Pf*ATP4 itself, rather than secondary molecular targets, triggers an influx of Na^+^ from the extracellular environment, which in turn prevents merozoite egress.

## 2 Materials and methods

### 2.1 Parasite lines and culture

Asexual blood-stage *P. falciparum* parasites were cultured according to established procedures (Trager and Jensen, 1976) in human RBCs (Australian Red Cross Blood Bank) diluted to 4% haematocrit in RPMI-1640 medium (Sigma-Aldrich) supplemented with 25 mM HEPES (Gibco), 0.37 mM hypoxanthine (Sigma-Aldrich), 31.25 μg/mL gentamicin (Gibco), 0.5% (w/v) AlbuMAX II (Gibco) and 0.2% (w/v) NaHCO_3_ (Thermo Scientific). Parasite strains utilised included wild-type 3D7 parasites and 3D7 parasites with a S374R mutation in *Pf*ATP4, which confers resistance to the *Pf*ATP4 inhibitor MB14 (MB14R^S374R^) (Gilson et al., 2019). To generate MB14R^S374R^_Hyp1-Nluc parasites, 100 μg of a pEF blasticidin deaminase drug selection plasmid containing Hyp1-Nluc (Azevedo et al., 2013) controlled by a *P. berghei ef1α* promoter was electroporated into uninfected RBCs (Deitsch et al., 2001; Hasenkamp et al., 2012). The electroporated RBCs were inoculated with MB14R^S374R^ trophozoite-infected RBCs and transfectant parasites were selected and maintained with 5 μg/mL blasticidin S (Sigma-Aldrich). 3D7_Hyp1-Nluc parasites had been generated by transfection previously (Azevedo et al., 2014) and were maintained using 2.5 nM WR99210 (Jacobus).

### 2.2 Compounds

MMV665878 (MolPort 002-647-671), MMV396719 (MolPort 002-620-441), MMV006239 (MolPort 002-099-136), MMV020136 (MolPort 000-136-457) and MMV020710 (MolPort 004-129-845) were purchased from MolPort and dissolved in dimethyl sulfoxide (DMSO). Cipargamin (a kind gift from Prof. Kiaran Kirk and Dr Adele Lehane, Australian National University) and WR99210 were dissolved in bicarbonate-free, AlbuMAX II-free RPMI medium. MB14 [synthesised by (Buskes et al., 2016)], Compound 1 (custom made), ML10 (LifeArc), zaprinast and artemisinin were dissolved in DMSO. E64, porcine heparin, chloroquine and blasticidin S were dissolved in Milli-Q water. Compounds were purchased from Sigma-Aldrich unless otherwise specified. In experiments in which each compound was tested at a single concentration, the concentration of DMSO used as a vehicle control matched the highest concentration of DMSO in any compound-treated well. Where compounds were serially diluted, the DMSO vehicle control concentration was equivalent to the highest DMSO concentration in the dilution series.

### 2.3 Experimental buffer preparation

The compositions of the Na^+^-based and K^+^-based buffers were adapted from the work of Pillai et al. (Pillai et al., 2013). The Na^+^-based buffer consisted of 102.7 mM NaCl, 5.4 mM KCl, 28.6 mM NaHCO_3_ and 5.64 mM Na_2_HPO_4_. The K^+^-based buffer consisted of 64.8 mM KCl, 28.6 mM KHCO_3_, 5.64 mM K_2_HPO_4_ and 84.3 mM sucrose. Both buffers were prepared in deionised water and the pH was adjusted to ~7.2 (comparable to the pH of supplemented RPMI medium, measured at 7.17) using 85% orthophosphoric acid. The osmolalities of the Na^+^-based and K^+^-based buffers were 267 mOsm/kg and 274 mOsm/kg respectively and were within the prescribed osmolality range for RPMI medium (267-277 mOsm/kg). Buffers were supplemented with 0.25% (w/v) AlbuMAX II prior to use.

### 2.4 Parasite synchronisation

Routine synchronisation was performed using 5% (w/v) D-sorbitol (Sigma-Aldrich) to selectively lyse trophozoites and schizonts (Lambros and Vanderberg, 1979). Where specified in the text, synchronous schizonts were obtained as follows: trophozoite-stage parasites that had been sorbitol synchronised on the preceding day were incubated with the reversible egress inhibitor ML10 (25-30 nM) overnight. The resultant culture, consisting of RBC-trapped schizonts, was washed twice, resuspended in drug-free supplemented RPMI medium and agitated at 50 rpm at 37°C for 2 h to allow parasites to egress and invade new RBCs. Unruptured schizonts were then lysed by sorbitol treatment, resulting in cultures of parasites aged 0-2 hours post invasion (hpi). Parasites were grown under normal culturing conditions for 40 h. Schizonts (aged 40-42 hpi) were then harvested by Percoll (GE Healthcare) gradient centrifugation (Rivadeneira et al., 1983) and used in assays. Due to the relatively inefficient invasion of MB14R^S374R^_Hyp1-Nluc parasites, a 3 h egress and invasion window was used for both 3D7_Hyp1-Nluc and MB14R^S374R^_Hyp1-Nluc parasites prior to experiments involving MB14R^S374R^_Hyp1-Nluc schizonts.

### 2.5 Parasite proliferation assay

Proliferation assays were performed as described previously (Gilson et al., 2019). Briefly, cultures of predominantly ring-stage parasites at 0.3% parasitaemia and 2% haematocrit were exposed to a 2-fold dilution series of compounds of interest during a 72-h incubation period at 37°C, after which the RBCs were lysed by freeze-thawing. Lactate dehydrogenase activity was then measured as an indicator of parasite biomass as described previously (Charnaud et al., 2018).

### 2.6 Bioluminescence assays

#### 2.6.1 Nanoluciferase egress and invasion assay

Conducted according to Dans et al. (2020) Percoll-purified schizonts at 1-2% parasitaemia and 1% haematocrit were incubated with serially diluted compounds of interest in 96-well U-bottom plates for 4 h. Supernatant was then harvested and 5 μL supernatant from each well was dispensed into 96-well luminometer plates. 45 μL of Nano-Glo assay reagent (consisting of Nano-Glo substrate (1:1000) in 1×Nano-Glo lysis buffer (Promega), diluted from 5× in Milli-Q water) was injected into each well of the luminometer plates and the bioluminescent signal intensity was immediately measured in relative light units (RLU) using a CLARIOstar luminometer (BMG Labtech). Any remaining unruptured schizonts in the U-bottom plates were lysed by sorbitol treatment to preserve only the ring-stage parasites that had developed during the 4 h treatment window. Pellets were washed twice and resuspended in drug-free supplemented RPMI medium and plates were incubated for a further 24 h. Pellets were then resuspended and 5 μL culture from each well was dispensed into luminometer plates. 45 μL Nano-Glo assay reagent was added to each well. After ~4 min incubation in the dark, the bioluminescent signal intensity of the culture samples (cells and medium, with parasites and RBCs lysed by the assay reagent to release intracellular nanoluciferase) was measured. The RLU of the culture samples provided an estimate of parasite biomass, thus indicating the success of invasion during the 4 h drug treatment window. A background control consisting of triplicate aliquots of Percoll-purified, drug-free schizont culture that were stored at 4°C during the 4 h egress and invasion window was included as described previously (Dans et al., 2020). For egress measurements, the background control accounted for nanoluciferase release during the 4 h egress and invasion window due to cell leakage. For invasion measurements, the background control accounted for the contribution to the final parasite biomass of ring-stage parasites that were retained during Percoll purification.

Percentage egress and invasion were calculated as follows, using the RLU of the supernatant or resuspended culture, respectively:


$$\frac{Mean\;RLU_{drug\;treated}-Mean\;RLU_{background}}{Mean\;RLU_{vehicle\;control}-Mean\;RLU_{background}}\times 100\%$$

In a modification of this assay, compounds were serially diluted in Na^+^-based and K^+^-based buffers, supplemented with 0.25% (w/v) AlbuMAX II, in 96-well U-bottom plates. Percoll-purified 3D7_Hyp1-Nluc schizonts were resuspended in the two supplemented buffers immediately prior to assays and added to the wells that contained compounds diluted in the corresponding buffer. As egress only (not invasion) was measured, the assay was terminated after the 4 h drug treatment window.

#### 2.6.2 Ring-stage growth inhibition assay

Cultures of early ring-stage 3D7_Hyp1-Nluc parasites (~0-4 hpi) were treated with sorbitol to lyse residual schizonts and resultant RBC pellets were washed in supplemented RPMI medium. In triplicate wells of 96-well U-bottom plates, ring-stage parasites at 2.5% parasitaemia and 1% haematocrit were treated with *Pf*ATP4 inhibitors (at a concentration ~10× higher than the IC_50_ for proliferation, corresponding to 0.7 μM MMV665878, 3.2 μM MMV396719, 2 μM MMV006239, 3 μM MMV020136, 0.6 μM MMV020710 and 10 nM cipargamin), chloroquine (~5×IC_50_; 75 nM), artemisinin (~8×IC_50_; 25 nM) or Compound 1 (~10×IC_50_; 4 μM). Triplicate wells containing 0.04% (v/v) DMSO were included as a vehicle control. After 4 h incubation at 37°C, drugs were removed by washing three times and pellets were resuspended in drug-free supplemented RPMI medium. Plates were incubated for a further 24 h, until parasites were ~24-28 hpi. Parasite biomass was then quantified by measuring the bioluminescent signal intensity of samples of resuspended culture, as in the nanoluciferase egress and invasion assay, and normalised to the vehicle control.

#### 2.6.3 Purified merozoite invasion assay

Adapted from the work of Boyle et al. and Wilson et al. (Boyle et al., 2010b; Wilson et al., 2013; Wilson et al., 2015) Briefly, 3D7_Hyp1-Nluc schizonts (~38-44 hpi) were concentrated using magnetic-activated cell sorting columns (Miltenyi Biotec) and incubated with the cysteine protease inhibitor E64 (10 µM) for ~4-6 h until discrete merozoites were visible inside the majority of schizonts. Approximately 30 min prior to the completion of this incubation period, test compounds and uninfected RBCs were dispensed into triplicate wells of a 96-well U-bottom plate. Schizonts were then mechanically ruptured by passage through a 1.2 µm syringe filter and the released merozoites were immediately added to all wells to achieve a final haematocrit of 1%. *Pf*ATP4 inhibitors and chloroquine were administered at the concentrations used in the ring-stage growth inhibition assay and heparin was administered at 100 µg/mL (Boyle et al., 2010a; Dans et al., 2020). 0.032% (v/v) DMSO was included as a vehicle control. The plate was then agitated at 300 rpm at 37°C for 30 min to allow merozoites to invade RBCs. Pellets were washed three times, resuspended in drug-free supplemented RMPI medium and incubated at 37°C for 24 h. Parasite biomass was then quantified as in the nanoluciferase egress and invasion assay and normalised to the vehicle control.

#### 2.6.4 Schizont rupture time-course assays

Synchronous cultures of 3D7_Hyp1-Nluc schizonts aged 40-42 hpi were concentrated by Percoll gradient centrifugation. In triplicate wells of 96-well U-bottom plates, 100 μL samples of schizont culture at 2% parasitaemia and 1% haematocrit were treated with *Pf*ATP4 inhibitors or Compound 1 at the concentrations used in the ring-stage growth inhibition assay. Triplicate wells containing 0.2% (v/v) DMSO were included as a vehicle control. An identical 96-well plate was set up for each timepoint (10, 20, 30, 40, 60, 120 and 240 min). 100 μL drug-free schizont culture was dispensed into triplicate wells of a separate plate, labelled t=0. The “t=0” plate was centrifuged at 200 g for 4 min and cell-free supernatant was collected. All other plates were incubated at 37°C. At each timepoint, one plate was removed from the incubator and supernatant collected as described for t=0. Supernatant was refrigerated at 4°C until the end of the assay. Schizont rupture was quantified by measuring the bioluminescent signal intensity of nanoluciferase in the harvested supernatant, as in the nanoluciferase egress and invasion assay, and normalised to the vehicle control at the final timepoint.

In a modification of this assay, Compound 1 and each of the *Pf*ATP4 inhibitors were administered to Percoll-purified schizonts at 2% parasitaemia and 1% haematocrit in six wells per drug. An additional three wells contained the 0.2% (v/v) DMSO vehicle control and three wells contained drug-free schizont culture. Immediately prior to incubation, 2 μL of 3.5 mM zaprinast was rapidly injected into the three drug-free wells and into three of the six wells containing each test compound to achieve a final concentration of 70 μM. Supernatant was collected and schizont rupture quantified as described above after 0, 20, 40 and 60 min.

In a second modification of this assay, 3D7_Hyp1-Nluc trophozoites aged ~30-34 hpi at >2% parasitaemia were exposed to 25 nM ML10 for ~15 h. ML10 was removed from the resultant RBC-trapped schizont culture by washing twice in supplemented RPMI medium. To reduce the time between ML10 washout and drug administration, and as ML10 prevents egress and subsequent ring formation, the schizonts were not Percoll-purified. Schizonts at 2% parasitaemia and 1% haematocrit were immediately exposed to *Pf*ATP4 inhibitors, Compound 1 or ML10 (25 nM) in triplicate wells of 96-well U-bottom plates. Supernatant was collected and schizont rupture quantified as described above after 0, 10, 20, 40, 60 and 120 min.

#### 2.6.5 Trophozoite lysis assay

Adapted from the work of Gilson et al. (Gilson et al., 2019) *Pf*ATP4 inhibitors, artemisinin and DMSO were diluted in Na^+^-based and K^+^-based buffers, supplemented with 0.25% (w/v) AlbuMAX II, and in supplemented RPMI medium and dispensed into 96-well U-bottom plates. 3D7_Hyp1-Nluc trophozoites aged ~26-32 hpi at 3% parasitaemia were resuspended in each of the three solutions and added to wells containing the corresponding solution, to achieve a final haematocrit of 1% in 100 μL per well. The following compounds were administered: ~5×IC_50_, ~10×IC_50_ and ~20×IC_50_ of MMV665878 (0.375 μM, 0.75 μM and 1.5 μM, respectively), MMV396719 (1.25 μM, 2.5 μM, 5 μM), MMV006239 (1 μM, 2 μM, 4 μM), MMV020136 (1.625 μM, 3.25 μM, 6.5 μM), MMV020710 (0.5 μM, 1 μM, 2 μM) and artemisinin (15 nM, 30 nM, 60 nM). A separate 0.05% (v/v) DMSO vehicle control was also prepared in each of the three solutions. Plates were incubated at 37°C for 4 h. Pellets were then resuspended by pipetting and 5 μL resuspended culture from each well was aliquoted into 96-well luminometer plates. U-bottom plates were then centrifuged at 200 g for 4 min and 5 μL cell-free supernatant from each well was aliquoted into luminometer plates. The bioluminescent signal intensities of culture lysates and cell-free supernatant samples were measured as in the nanoluciferase egress and invasion assay.

Percentage lysis was calculated as follows:


$$\frac{Mean\;RLU_{supernatant}}{Mean\;RLU_{culture\;lysate}}\times 100\%$$

Compound-dependent percentage lysis was calculated by subtracting the percentage lysis in DMSO-treated wells from the percentage lysis in drug-treated wells.

### 2.7 Graphical and statistical analysis

Graphs were produced and statistical analysis conducted using GraphPad Prism (version 9.1.1). Fitted curves were plotted and IC_50_ values computed using the model “log(inhibitor) vs. response – variable slope (four parameters)”. The unpaired Student’s t-test and one-way analysis of variance (ANOVA) followed by Dunnett’s and Tukey’s multiple comparisons tests were applied as specified in the text and figure legends, using a significance level of 0.05 throughout.

## 3 Results

### 3.1 *Pf*ATP4 inhibitors from the MMV Malaria Box and Pathogen Box have reduced potency against *Pf*ATP4-mutant parasites

**Figure 1:**
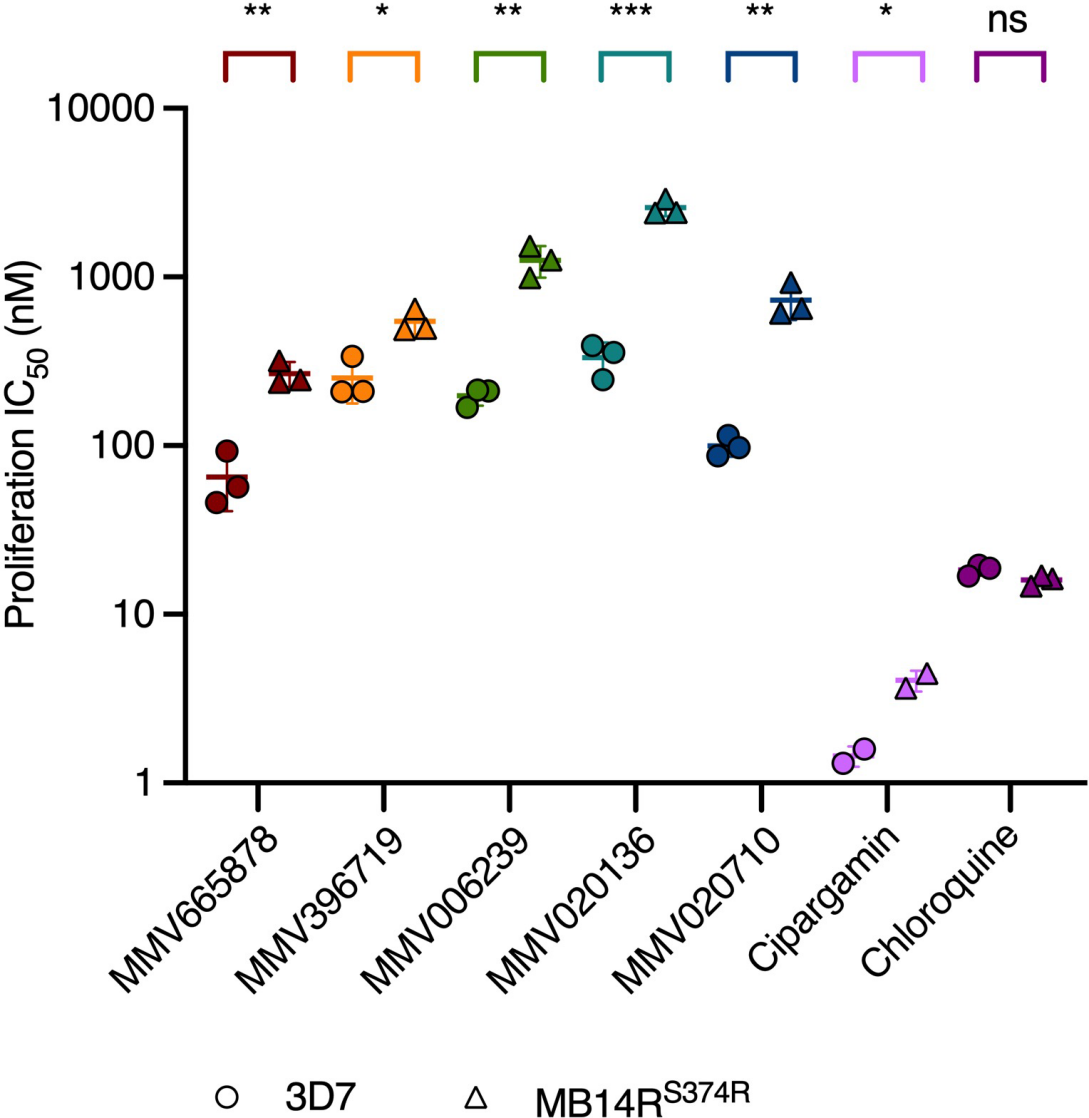
*Pf*ATP4-mutant MB14R^S374R^ parasites are cross resistant to multiple *Pf*ATP4 inhibitors but not to the mechanistically unrelated antimalarial chloroquine. MB14R^S374R^ parasites (triangles) and the parental 3D7 parasite line (circles) were treated with a 2-fold dilution series of each test compound and incubated for 72 h. Lactate dehydrogenase activity was then measured as an indicator of parasite biomass and the IC_50_ for proliferation was estimated. Lines and error bars indicate the mean IC_50_ ± SD of three biological replicates (except for cipargamin, for which two biological replicates were conducted), each consisting of technical triplicates. The statistical significance of the difference between the mean IC_50_s for the two parasite lines was calculated using the unpaired Student’s t-test (ns: not significant, *p<0.05, **p<0.01, ***p<0.001). IC_50_ values, fold differences and p-values are listed in **Supplementary Table 1**.

The Malaria Box compounds MMV665878 and MMV396719 and the Pathogen Box compounds MMV006239, MMV020136 and MMV020710 were classified previously as likely *Pf*ATP4 inhibitors based on their effects on Na^+^ and pH homeostasis in asexual blood-stage *P. falciparum* (**Supplementary Figure 1**). Specifically, all five compounds were found to induce intra-parasitic alkalinisation and Na^+^ accumulation *in vitro* (Lehane et al., 2014; Dennis et al., 2018b). Another identifying feature of the *Pf*ATP4 inhibitors is that parasites selected for resistance to one *Pf*ATP4 inhibitor are often cross-resistant to others (Jiménez-Díaz et al., 2014; Lehane et al., 2014; Flannery et al., 2015; Dennis et al., 2018b; Gilson et al., 2019). We investigated whether our five compounds also exhibited this property by measuring their activities against parasites selected for resistance to the *Pf*ATP4 inhibitor MB14, which carry a S374R mutation in the *pfatp4* gene (MB14R^S374R^) (Gilson et al., 2019). After a 72 h drug exposure window, the IC_50_ of each compound was estimated by measuring lactate dehydrogenase activity as a proxy for parasite proliferation (Makler and Hinrichs, 1993). Two well-characterised *Pf*ATP4 inhibitors were also tested in this assay: cipargamin, a spiroindolone antimalarial currently in clinical trials for severe malaria (Rottmann et al., 2010; National Library of Medicine (U. S.), 2022b), and MB14 (**Supplementary Figure 1**). The MB14R^S374R^ parasite line was significantly less sensitive than the wild-type 3D7 line to cipargamin (2.8-fold increase in IC_50_; p<0.05; unpaired Student’s t-test; **Figure 1** and **Supplementary Table 1**) and grew to ~85% of vehicle control levels at the highest concentration of MB14 administered in this assay (10 μM) (**Supplementary Figure 2**). The MB14R^S374R^ parasite line was significantly cross-resistant to all five MMV compounds (p<0.03; **Figure 1** and **Supplementary Table 1**). The fold increase in IC_50_ in the MB14R^S374R^ parasites relative to the parental line ranged from 2.2 (for MMV396719) to 7.8 (MMV020136). In contrast, the IC_50_ for a mechanistically unrelated antimalarial, chloroquine, was comparable for the two parasite lines (p=0.084). These data are consistent with the five MMV compounds sharing a *Pf*ATP4-dependent MoA.

### 3.2 Investigating the timing of action of *Pf*ATP4 inhibitors during blood-stage merozoite egress and invasion using a bioluminescent reporter enzyme

In 2018, Subramaniam et al. used flow cytometry and microscopy to identify inhibitors of the schizont-to-ring transition in the MMV Malaria Box, classifying MMV396719 and MMV665878 as an egress inhibitor and an invasion inhibitor respectively (Subramanian et al., 2018). In a 2020 screen of the MMV Pathogen Box, Dans et al. classified MMV006239 as an egress inhibitor and MMV020136 and MMV020710 as invasion inhibitors (Dans et al., 2020). We interrogated these findings using 3D7 parasites transfected with a bioluminescent nanoluciferase enzyme fused to the N-terminal region of the exported Hyp1 protein (3D7_Hyp1-Nluc) (Azevedo et al., 2014). This fusion protein contains the *Plasmodium* export element (PEXEL) motif from Hyp1 and is therefore exported into the RBC compartment and released into the culture medium upon schizont rupture. Egress can therefore be quantified by exposing samples of cell-free culture medium to the furimazine substrate of nanoluciferase (Nano-Glo) and measuring the resultant bioluminescent signal (Hall et al., 2012; Azevedo et al., 2013). To quantify invasion, infected RBCs were chemically lysed to release intracellular nanoluciferase and exposed to Nano-Glo substrate. The bioluminescent signal intensity of the lysate was proportional to parasite biomass and therefore indicated the fraction of RBCs that were successfully invaded.

A potential explanation for the inconsistent effects of *Pf*ATP4 inhibitors during egress and invasion, as reported in the literature, was that these inhibitors blocked egress at certain concentrations and invasion at others. We investigated this possibility by generating concentration-response curves using a recently-developed assay (Dans et al., 2020) referred to herein as the “nanoluciferase egress and invasion assay”. Here, 3D7_Hyp1-Nluc schizonts were added to uninfected RBCs in the presence of serially diluted inhibitors. After 4 h, cell-free culture medium was harvested and used to quantify egress, and unruptured schizonts were lysed by sorbitol treatment (Lambros and Vanderberg, 1979), leaving intact any ring-stage parasites that had developed during the 4 h treatment window. Since young ring-stage parasites express negligible levels of nanoluciferase (Azevedo et al., 2013), invasion could not be measured immediately. The ring-infected RBCs were therefore resuspended in drug-free medium and incubated for 24 h to allow nanoluciferase expression to reach detectable levels. The bioluminescent signal of chemically lysed trophozoite cultures was then measured. Compound 1 (an inhibitor of *Pf*PKG which stalls the parasite’s cell-cycle minutes before egress is due to take place (Gurnett et al., 2002; Collins et al., 2013)) and heparin (Boyle et al., 2010a) were included as positive controls for egress and invasion inhibition, respectively. In this assay, Compound 1, an exclusive egress inhibitor, resembled a dual egress and invasion inhibitor as it caused the invasive merozoites to become trapped inside unruptured schizonts, resulting in minimal ring formation and therefore low parasite biomass after the 24 h drug-free incubation period (**Supplementary Figure 3A**). In contrast, heparin was found to permit egress at all concentrations tested but inhibited invasion in a concentration-dependent manner (**Supplementary Figure 3B**). Due to the assay design, new rings were potentially exposed to test compounds for up to 4 h, meaning that drugs with anti-parasitic activity against rings may have been erroneously classified as invasion inhibitors. To exclude this possibility, we treated early ring-stage parasites with ~10×IC_50_ of *Pf*ATP4 inhibitors for 4 h (**Supplementary Figure 4**). Drugs were then removed and parasites were incubated for 24 h, until the trophozoite stage. The parasitaemia was then quantified by measuring the nanoluciferase signal of chemically lysed trophozoite cultures. Compound 1, which is inactive against intra-erythrocytic parasite growth (Taylor et al., 2010), served as a negative control; the antimalarials chloroquine and artemisinin disrupt ring-stage development (Wilson et al., 2013; Shivapurkar et al., 2018) and served as positive controls. All *Pf*ATP4 inhibitors behaved similarly to Compound 1 in this assay (p≥0.6; one-way ANOVA with Dunnett’s multiple comparisons test), indicating that these compounds do not arrest ring-stage development or that their effect is reversible.

**Figure 2:**
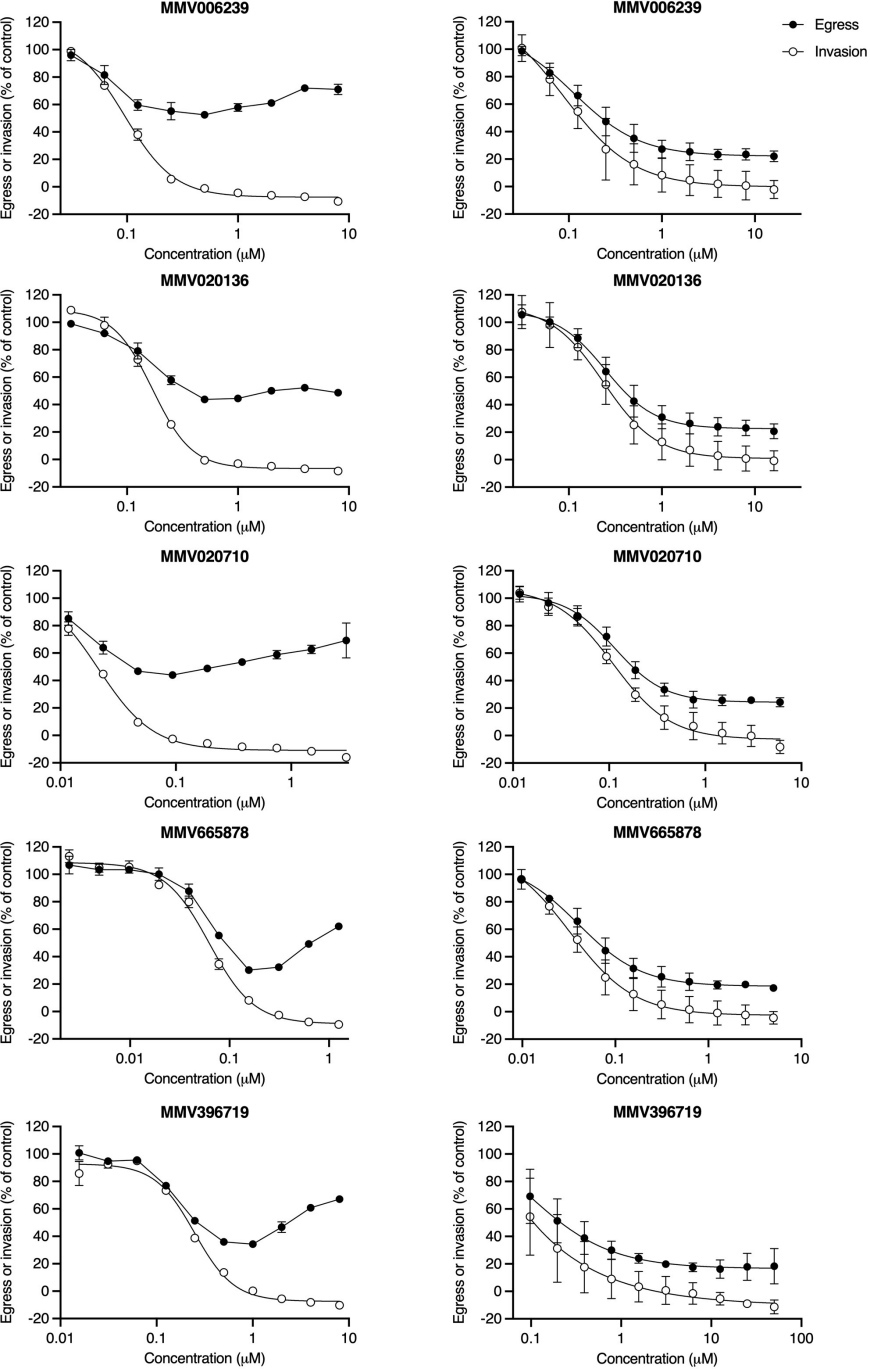
*Pf*ATP4 inhibitors induce cell rupture when administered to cultures of schizonts with a wide age range but prevent the rupture of synchronous late-stage schizonts. In the “nanoluciferase egress and invasion assay”, 3D7_Hyp1-Nluc schizonts were exposed to a 2-fold dilution series of *Pf*ATP4 inhibitors. Egress was quantified after 4 h by measuring the bioluminescent signal intensity of nanoluciferase released into the extracellular medium. Drugs were then removed and unruptured schizonts were lysed by sorbitol treatment. Invasion was quantified 24 h later by chemically lysing the resultant trophozoite cultures and measuring the total nanoluciferase signal of the lysate. When the “nanoluciferase egress and invasion assay” was conducted using schizonts with a wide age range (~35-42hpi; left-hand column), high concentrations of *Pf*ATP4 inhibitors did not inhibit nanoluciferase release but did reduce subsequent invasion. For each compound, data obtained in a single experiment are shown as an illustrative example of those obtained in at least two independent experiments (**Supplementary Figure 5**). Points and error bars represent the mean ± SD of technical duplicates or triplicates. When this assay was conducted using late-stage schizonts with a narrow age range (40-42 hpi; right-hand column), *Pf*ATP4 inhibitors caused concentration-dependent inhibition of egress and invasion and did not block invasion independently of egress at any concentration. Points and error bars represent the mean ± SD of three biological replicates, each consisting of technical duplicates. All values have been normalised to the DMSO vehicle control.

The “nanoluciferase egress and invasion assay” was initially conducted using 3D7_Hyp1-Nluc parasites that had been routinely synchronised by sorbitol treatment (Lambros and Vanderberg, 1979) during the preceding growth cycles. Sorbitol induces osmotic lysis of schizonts and trophozoites but preserves ring-stage parasites (~0-24 hpi) (Wagner et al., 2003; Counihan et al., 2017) and is therefore a relatively imprecise synchronisation tool which results in mixed populations of early/mid/late-stage schizonts at the time of the assay. In this context, low concentrations of *Pf*ATP4 inhibitors moderately reduced nanoluciferase release relative to the vehicle control (**Figure 2**, left-hand column and **Supplementary Figure 5**). This trend was reversed at higher concentrations, with increasing drug concentration corresponding to increasing release of nanoluciferase, such that the extracellular bioluminescent signal approached (or, in some cases, exceeded) that measured in vehicle control-treated parasite samples. Despite pronounced inter-assay variability in the egress curves, the MMV compounds appeared to consistently reduce subsequent invasion in a concentration-dependent manner.

### 3.3 *Pf*ATP4 inhibitors do not directly inhibit invasion

**Figure 3:**
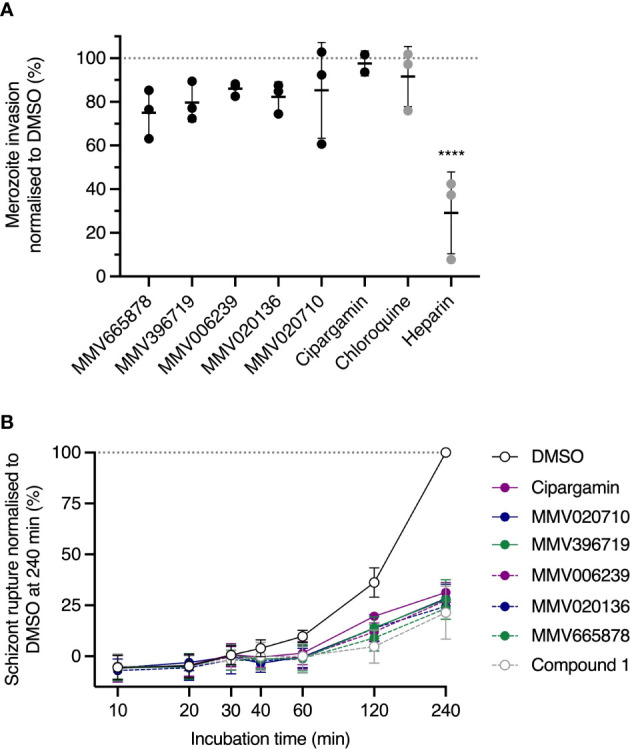
*Pf*ATP4 inhibitors dysregulate 3D7_Hyp1-Nluc merozoite egress but do not directly target merozoite invasion. **(A)** Mechanically purified merozoites were allowed to invade RBCs for 30 min in the presence of ~10×IC_50_ of *Pf*ATP4 inhibitors (0.7 μM MMV665878, 3.2 μM MMV396719, 2 μM MMV006239, 3 μM MMV020136, 0.6 μM MMV020710, 10 nM cipargamin). Compounds were then removed and invasion was quantified 24 h later by measuring the nanoluciferase signals of the trophozoite cultures (cells and medium, with host cells and parasites chemically lysed to release intracellular nanoluciferase). Chloroquine (75 nM; ~5×IC_50_) and heparin [100 µg/mL (Boyle et al., 2010a; Dans et al., 2020)] served as negative and positive controls for invasion inhibition, respectively. Values were normalised to the DMSO control. **(B)** Synchronised schizonts aged 40-42 hpi were exposed to ~10×IC_50_ of *Pf*ATP4 inhibitors or the *Pf*ATP4-independent egress inhibitor Compound 1. Egress was quantified by periodically measuring the bioluminescent signal intensity of nanoluciferase released into the growth medium. The bioluminescent signal was normalised to that measured in DMSO-treated wells at the final timepoint after subtracting the baseline bioluminescence (measured at 0 min). Points and error bars represent the mean ± SD of three biological replicates (except for cipargamin in **(A)**, for which n=2), each consisting of technical triplicates. Statistical analysis was performed *via* one-way ANOVA with Dunnett’s multiple comparisons test for comparison to the negative control, chloroquine. ****p<0.0001. No asterisk indicates not significant.

An inherent limitation of the “nanoluciferase egress and invasion assay” is that it does not distinguish between two distinct mechanisms of invasion inhibition. The first mechanism corresponds to compounds that act on unruptured schizonts to reduce the invasive capacity of released merozoites and the second mechanism applies to compounds that directly target free merozoites or disrupt the interaction between merozoites and uninfected RBCs. We therefore sought to clarify the timing of action of our chosen *Pf*ATP4 inhibitors by testing these compounds for their capacity to inhibit the invasion of purified merozoites (**Figure 3A**) (Boyle et al., 2010b; Wilson et al., 2013) Briefly, magnet-purified schizonts were incubated with the cysteine protease inhibitor E64 (Glushakova et al., 2009), which prevents rupture of the host cell membrane, until discrete merozoites were visible inside the schizonts as determined by Giemsa-stained smears. The mature schizonts were then mechanically ruptured to release merozoites, which were immediately added to uninfected RBCs and allowed to invade for 30 min in the presence of ~10×IC_50_ of *Pf*ATP4 inhibitors. The inhibitors were then removed and the infected RBCs were resuspended in drug-free medium and incubated for 24 h. Invasion was quantified as described above and the invasion efficiencies in the presence of *Pf*ATP4 inhibitors ranged from 78.5 ± 6.1% (for MMV665878; mean ± SD) to 97.6 ± 5.7% (cipargamin). No *Pf*ATP4 inhibitor differed significantly from the negative control antimalarial chloroquine, which does not affect invasion (Burns et al., 2019) (p>0.2; one-way ANOVA with Dunnett’s multiple comparisons test). Conversely, a known invasion inhibitor, heparin (Boyle et al., 2010a), reduced invasion to 29.6 ± 18% (p<0.0001). These data indicate that *Pf*ATP4 inhibitors neither block parasite invasion directly nor prevent the conversion of merozoites to rings.

### 3.4 *Pf*ATP4 inhibitors block merozoite egress from synchronous late-stage schizonts

*P. falciparum* replicates by schizogony, during which successive, asynchronous nuclear divisions are followed by a single, synchronous cytokinetic division (Rudlaff et al., 2020). This results in a segmented schizont containing ~16-32 merozoites (Salmon et al., 2001). The precise temporal regulation of merozoite egress is therefore crucial, as early egress would result in the release of immature, non-invasive merozoites. As it was possible that *Pf*ATP4 inhibition prevented invasion indirectly by accelerating or prematurely triggering egress, we investigated this by monitoring the trajectory of egress during a 4 h drug treatment period. Prior to the assay, 3D7_Hyp1-Nluc parasites (which have an asexual cell cycle of ~44 h in our laboratory) were synchronised to a 2 h age window and grown to the late schizont stage (40-42 hpi). Schizonts were then exposed to ~10×IC_50_ of *Pf*ATP4 inhibitors and cell-free medium was harvested and the nanoluciferase signal intensity was measured after 10, 20, 30, 40, 60, 120 and 240 min (**Figure 3B**). Compound 1 and a DMSO vehicle control served as positive and negative controls for egress inhibition, respectively. By 120 min, all *Pf*ATP4 inhibitors except cipargamin had significantly reduced egress relative to DMSO (p ≤ 0.01; one-way ANOVA with Tukey’s multiple comparisons test). For cipargamin, the inhibition of egress reached significance at 240 min (p<0.0001). No *Pf*ATP4 inhibitor differed significantly from Compound 1 at any time point. The data indicate that the extent and trajectory of egress are comparable during treatment with *Pf*ATP4 inhibitors or Compound 1 and thus exclude the possibility that *Pf*ATP4 inhibition accelerates merozoite egress. The inhibition of egress measured in the nanoluciferase-based assay was subsequently validated by examination of Giemsa-stained thin blood smears prepared after treating synchronous late-stage schizonts with ~10×IC_50_ of *Pf*ATP4 inhibitors for 4 h, by which time vehicle control-treated cultures consisted predominantly of ring-stage parasites (**Supplementary Figure 6**). The *Pf*PKG inhibitors Compound 1 and ML10 and the cysteine protease inhibitor E64, which acts downstream of ML10 and Compound 1 (Glushakova et al., 2009; Dans et al., 2020), served as positive controls for egress inhibition. Whereas E64-treated schizonts tended to rupture during smearing, the five MMV compounds resembled Compound 1 and ML10 in that they produced RBC-trapped schizonts which remained intact during smearing.

As the egress inhibition observed in schizont rupture time-course assays and in Giemsa-stained smears contradicted the apparent “enhanced rupture” phenotype seen in the “nanoluciferase egress and invasion assay”, we repeated the latter assay using 3D7_Hyp1-Nluc parasites that had been synchronised to a 2 h age range and grown to the late schizont stage (40-42 hpi). The treated parasite populations now consisted primarily of well-segmented schizonts, with minimal early/mid-stage schizonts. In this context, the five MMV compounds resembled Compound 1 (**Supplementary Figure 3A**), reducing egress and invasion in a concentration-dependent manner without exclusively inhibiting invasion at any concentration (**Figure 2**, right-hand column). However, whereas the mean bottom plateaus of the egress and invasion curves for Compound 1 did not differ significantly (p=0.4; unpaired Student’s t-test), the invasion concentration-response curve generated by each of the five MMV compounds reached a significantly lower bottom plateau than the corresponding egress curve (p ≤ 0.03).

Taken together, these data suggest that the effects of *Pf*ATP4 inhibition on schizont rupture depend heavily on schizont age. Specifically, the *Pf*ATP4 inhibitors appeared to block egress from late schizont-infected RBCs; however, in the presence of younger schizonts, high drug concentrations enhanced nanoluciferase release, possibly by inducing lysis of infected RBCs (examined in a later section).

### 3.5 *Pf*ATP4 inhibitors target egress by interacting with *Pf*ATP4 rather than secondary molecular targets

#### 3.5.1 A mutation in *Pf*ATP4 confers partial resistance to egress inhibition by *Pf*ATP4 inhibitors

**Figure 4:**
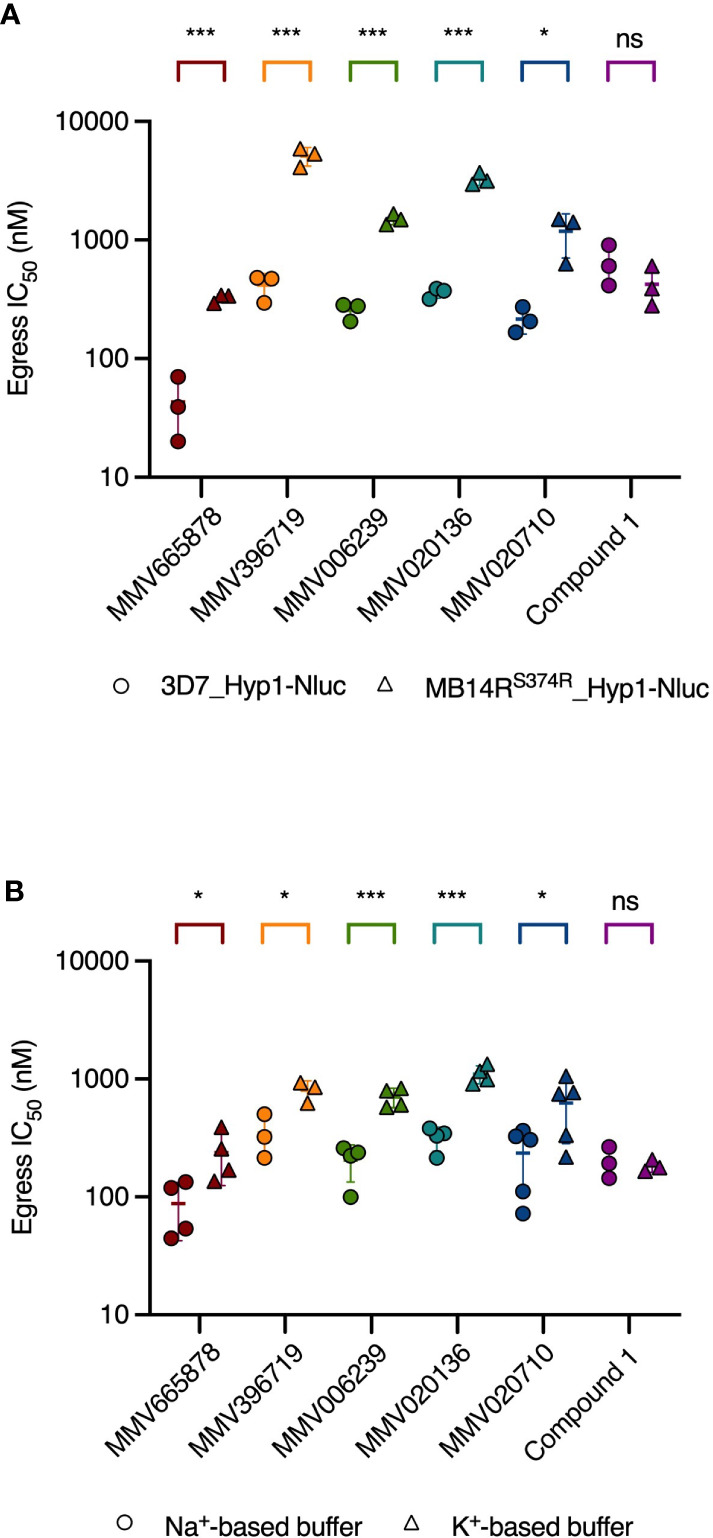
The potency of egress inhibition by *Pf*ATP4 inhibitors is reduced in parasites with a resistance-conferring mutation in *Pf*ATP4 and in wild-type parasites treated in a Na^+^-depleted buffer. **(A)** *Pf*ATP4-mutant MB14R^S374R^_Hyp1-Nluc parasites (triangles) are less sensitive than 3D7_Hyp1-Nluc parasites (circles) to egress inhibition by the *Pf*ATP4 inhibitors. The potency of the mechanistically unrelated egress inhibitor Compound 1 does not differ significantly between the two parasite lines. **(B)** 3D7_Hyp1-Nluc parasites are less sensitive to egress inhibition by *Pf*ATP4 inhibitors when suspended in a Na^+^-depleted, K^+^-based buffer (triangles) than when suspended in a buffer with a Na^+^ concentration equal to that of standard RPMI medium (circles). For Compound 1, the IC_50_ for egress inhibition (derived from the graphs shown in **Supplementary Figure 8**) does not differ significantly between the two buffers. Synchronised late-stage schizonts [40-43 hpi **(A)** or 40-42 hpi **(B)**] were treated with a 2-fold dilution series of each test compound and incubated for 4 h. Egress was then quantified by measuring the bioluminescent signal intensity of nanoluciferase released into the growth medium. Lines and error bars indicate the mean IC_50_ ± SD of at least three biological replicates, each consisting of technical duplicates. The statistical significance of the difference between mean IC_50_s for was calculated using the unpaired Student’s t-test (ns: not significant, *p<0.05, ***p<0.001). IC_50_ values, fold differences and p-values are listed in **Supplementary Table 1**.

Since it was not clear why compounds that dysregulate Na^+^ and pH homeostasis in schizonts would block merozoite egress, it remained possible that our chosen *Pf*ATP4 inhibitors blocked egress by binding to secondary, as-yet unidentified target proteins. We investigated this possibility by comparing the potency with which these compounds inhibited egress in wild-type and *Pf*ATP4-mutant parasites. MB14R^S374R^ parasites were transfected with the Hyp1-Nluc reporter construct (MB14R^S374R^_Hyp1-Nluc) for use in nanoluciferase-based egress assays. MB14R^S374R^_Hyp1-Nluc and 3D7_Hyp1-Nluc parasites were synchronised to a 3 h age window and grown to the late schizont stage (40-43 hpi) before being treated with serially diluted *Pf*ATP4 inhibitors for 4 h. Egress was then quantified by measuring the nanoluciferase signal of the cell-free growth medium. Each of the five MMV compounds tested inhibited the egress of MB14R^S374R^_Hyp1-Nluc merozoites significantly less potently than that of 3D7_Hyp1-Nluc merozoites (p ≤ 0.025; unpaired Student’s t-test; **Figure 4A** and **Supplementary Table 1**). The fold difference in the egress IC_50_ between the two parasite lines ranged from 5.5 (for MMV020710) to 12 (MMV396719). The IC_50_ for a mechanistically unrelated egress inhibitor, Compound 1, was slightly but not significantly lower in MB14R^S374R^_Hyp1-Nluc parasites than in 3D7_Hyp1-Nluc parasites (0.66-fold difference; p=0.27). These data suggest that the five MMV compounds inhibit egress by a *Pf*ATP4-dependent mechanism and therefore that *Pf*ATP4 is likely important for merozoite egress.

#### 3.5.2 Replacement of extracellular Na^+^ with K^+^ reduces the egress-inhibitory potency of *Pf*ATP4 inhibitors

By disabling the parasite’s primary Na^+^ extrusion mechanism, *Pf*ATP4 inhibitors cause a rapid and immediate-onset rise in intra-parasitic Na^+^ levels (Spillman et al., 2013b; Lehane et al., 2014; Dennis et al., 2018b). If egress is blocked as a downstream consequence of Na^+^ accumulation, then preventing Na^+^ build-up by depleting the extracellular medium of Na^+^ should attenuate the inhibition of egress. Thus, by exposing schizonts to *Pf*ATP4 inhibitors in Na^+^-based and Na^+^-depleted media, a mechanistic link between *Pf*ATP4 inhibition and egress blockade may be inferred.

With this rationale, we prepared two buffers: one with a Na^+^ concentration approximately equal to that of standard RPMI medium and another in which Na^+^ had been isosmotically replaced by K^+^ and sucrose (Pillai et al., 2013). The MMV compounds and the control egress inhibitor Compound 1 were serially diluted in Na^+^-based or K^+^-based buffer and administered to 3D7_Hyp1-Nluc schizonts which had been resuspended in these buffers immediately prior to assays. After 4 h, the extracellular nanoluciferase signal was measured as an indicator of egress. When administered to late-stage schizonts that had been synchronised to a 2 h age window (40-42 hpi), all five MMV compounds inhibited egress in a concentration-dependent manner in both buffers, as did Compound 1. However, while the potency of Compound 1 was comparable across the two buffers (0.91-fold difference in IC_50_; p=0.67; unpaired Student’s t-test; **Figure 4B** and **Supplementary Table 1**), the five MMV compounds inhibited egress significantly less potently in the K^+^-based buffer than in the Na^+^-based buffer (p<0.05). The fold difference in egress IC_50_ in the K^+^-based buffer relative to the Na^+^-based buffer ranged from 2.3 (for MMV396719) to 3.5 (MMV020136).

**Figure 5:**
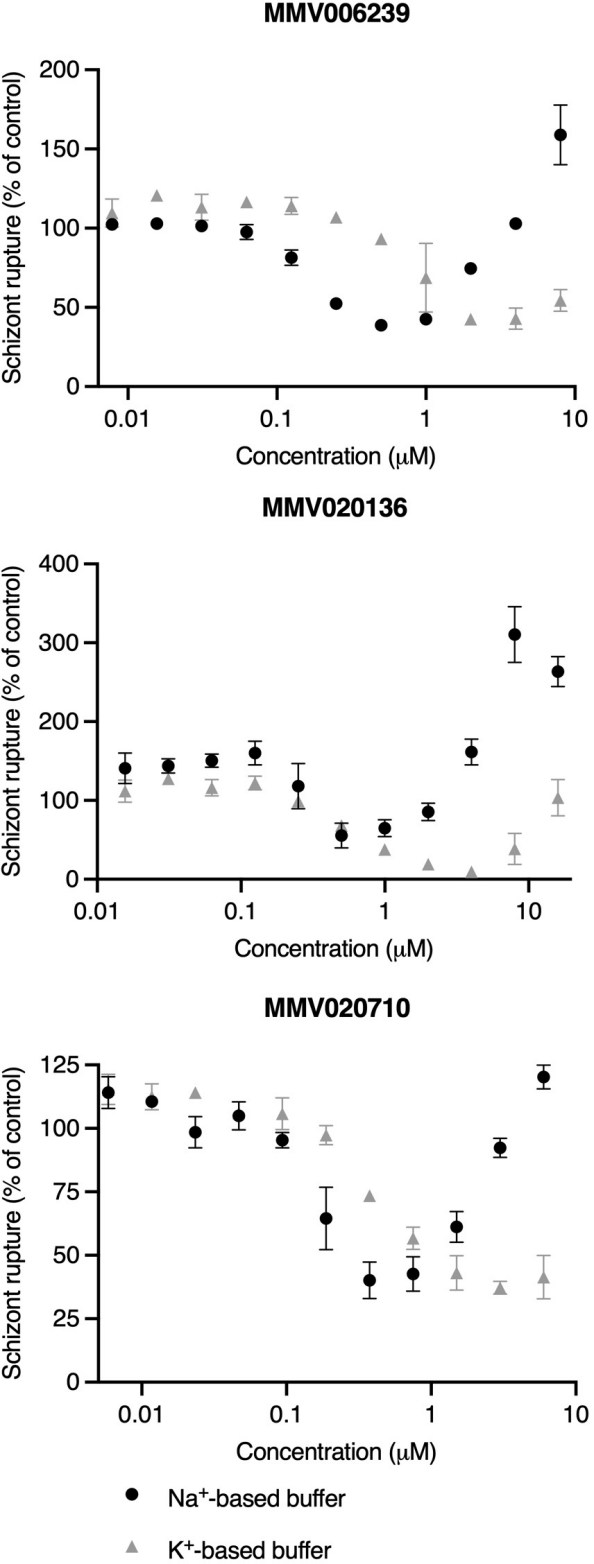
When administered to early/mid-stage schizonts, high concentrations of *Pf*ATP4 inhibitors promote nanoluciferase release in a Na^+^-dependent manner. Early/mid-stage schizonts (~36-40 hpi) that had not fully completed merozoite division were resuspended in a Na^+^-based buffer (black circles) or a Na^+^-depleted, K^+^-based buffer (grey triangles) and incubated with a 2-fold dilution series of *Pf*ATP4 inhibitors for 4 h. The bioluminescent signal intensity of nanoluciferase released into the buffer was then measured and values were normalised to the DMSO vehicle control. Lines and error bars indicate the mean ± SD of technical duplicates. Data obtained in a single experiment are shown as an illustrative example of those obtained in three independent experiments (**Supplementary Figure 7**).

Having observed that high concentrations of the MMV compounds tended to promote nanoluciferase release when administered to relatively asynchronous schizont populations in standard culture medium (**Figure 2**, left-hand column) but not when administered to synchronous late-stage schizonts (**Figure 2**, right-hand column), we proposed that *Pf*ATP4 inhibition induces Na^+^-dependent osmotic rupture of early/mid-stage schizonts that have not undergone complete merozoite segmentation. To validate this hypothesis, we repeated the above assay using schizont cultures that had been synchronised to a 2 h age window and grown to ~36-40 hpi prior to assays (**Figure 5** and **Supplementary Figure 7**). In this context, increasing concentrations of *Pf*ATP4 inhibitors tested initially corresponded to decreasing nanoluciferase release in both buffers, as anticipated. However, at high drug concentrations, nanoluciferase release increased with increasing drug concentrations. This effect occurred at a lower concentration, and to a greater extent, in Na^+^-based buffer than in K^+^-based buffer. In contrast, Compound 1 behaved as a concentration-dependent inhibitor of schizont rupture and nanoluciferase release in both buffers regardless of schizont age (**Supplementary Figure 8**).

### 3.6 *Pf*ATP4 inhibitors induce Na^+^-dependent lysis of infected RBCs

**Figure 6:**
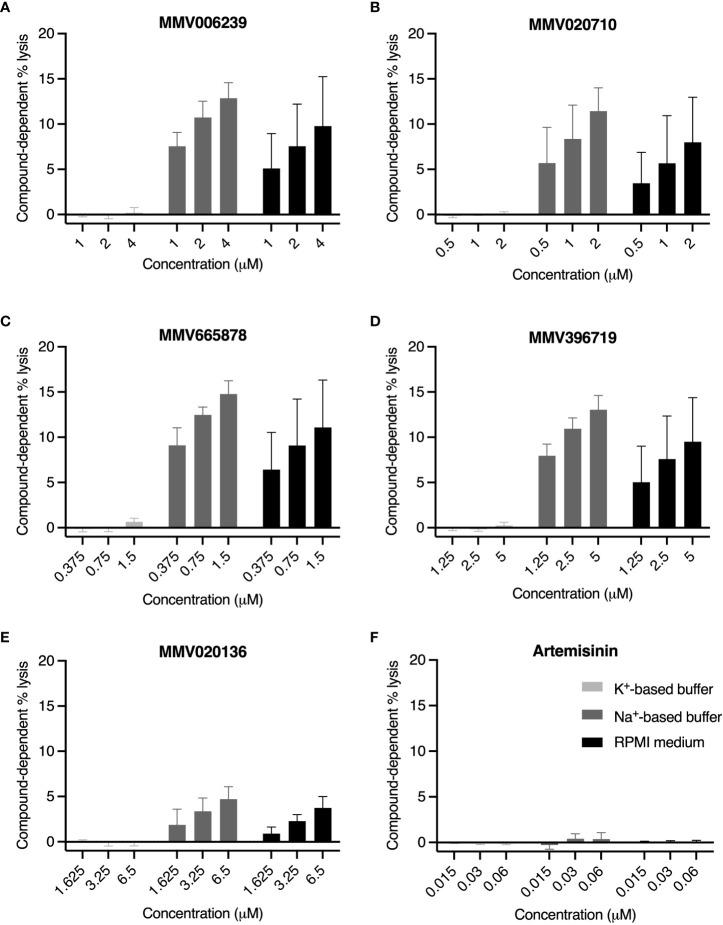
*Pf*ATP4 inhibitors induce Na^+^-dependent, concentration-dependent lysis of RBCs infected with 3D7_Hyp1-Nluc trophozoites. Trophozoites (~26-32 hpi) were resuspended in K^+^-based buffer (light grey bars), Na^+^-based buffer (dark grey bars) or standard RPMI medium (black bars). PfATP4 inhibitors **(A–E)** and the mechanistically unrelated antimalarial artemisinin **(F)** were diluted in the corresponding solutions and administered for 4 h at the concentrations specified (μM), corresponding to ~5×IC_50_, ~10×IC_50_ and ~20×IC_50_. Lysis was assessed by measuring the bioluminescent signal intensity of nanoluciferase released into the cell-free growth medium and expressing this as a percentage of the signal intensity of the total culture (cells and medium, with host and parasite cells chemically lysed). Compound-dependent lysis was then estimated by subtracting the percentage lysis measured in wells treated with the DMSO vehicle control. Graphs indicate the mean ± SD compound-dependent percentage lysis of four biological replicates, each consisting of technical duplicates or triplicates.

Several *Pf*ATP4 inhibitors have been reported to induce swelling and lysis of trophozoite- and schizont-infected RBCs (Jiménez-Díaz et al., 2014; Vaidya et al., 2014; Dennis et al., 2018a; Gilson et al., 2019), likely due to the osmotic influx of water that accompanies intra-parasitic Na^+^ accumulation. We therefore speculated that the “enhanced rupture” phenotype observed when high concentrations of *Pf*ATP4 inhibitors were added to minimally segmented schizonts or schizont populations spanning a broad age range was attributable to Na^+^-dependent osmotic lysis of the host cell. To test this hypothesis, we treated 3D7_Hyp1-Nluc trophozoites (~26-32 hpi) with *Pf*ATP4 inhibitors in K^+^-based buffer, Na^+^-based buffer and standard RPMI medium (**Figures 6A–E**). Drugs were administered at concentrations of ~5×, 10× and 20× the IC_50_, estimated using the lactate dehydrogenase growth assay. Artemisinin, a potent but non-lytic antimalarial (Gilson et al., 2019), served as a negative control (**Figure 6F**). After a 4 h drug treatment period, the growth medium was harvested and the extent of cell lysis was computed by measuring the nanoluciferase released into the medium and expressing this as a percentage of the total nanoluciferase content of the whole culture (medium and cells, with parasites and host cells chemically lysed to release nanoluciferase). The percentage lysis at 4 h in DMSO-treated samples was subtracted from the percentage lysis in drug-treated samples to yield the compound-dependent cell lysis.

In K^+^-based buffer, compound-dependent lysis was close to negligible for all MMV compounds, ranging from -0.16 ± 0.25% (for MMV396719 at 10×IC_50_; mean ± SD) to 0.66 ± 0.38% (MMV665878 at 20×IC_50_), and displayed no discernible concentration dependence. In Na^+^-based buffer and RPMI medium, the MMV compounds induced nanoluciferase release in a concentration-dependent manner. MMV665878 was the most lytic of the five compounds, causing 15 ± 1.5% lysis and 11 ± 5.2% lysis at 20×IC_50_ in Na^+^-based buffer and RPMI medium respectively. At all concentrations tested, the MMV compounds induced slightly but not significantly less lysis in RPMI medium than in Na^+^-based buffer (p>0.21; unpaired Student’s t-test). As anticipated, artemisinin caused minimal lysis in all conditions, consistent with the induced release of nanoluciferase being a consequence of *Pf*ATP4 inhibition.

### 3.7 *Pf*ATP4 inhibitors act upstream of *Pf*PKG to inhibit egress

**Figure 7:**
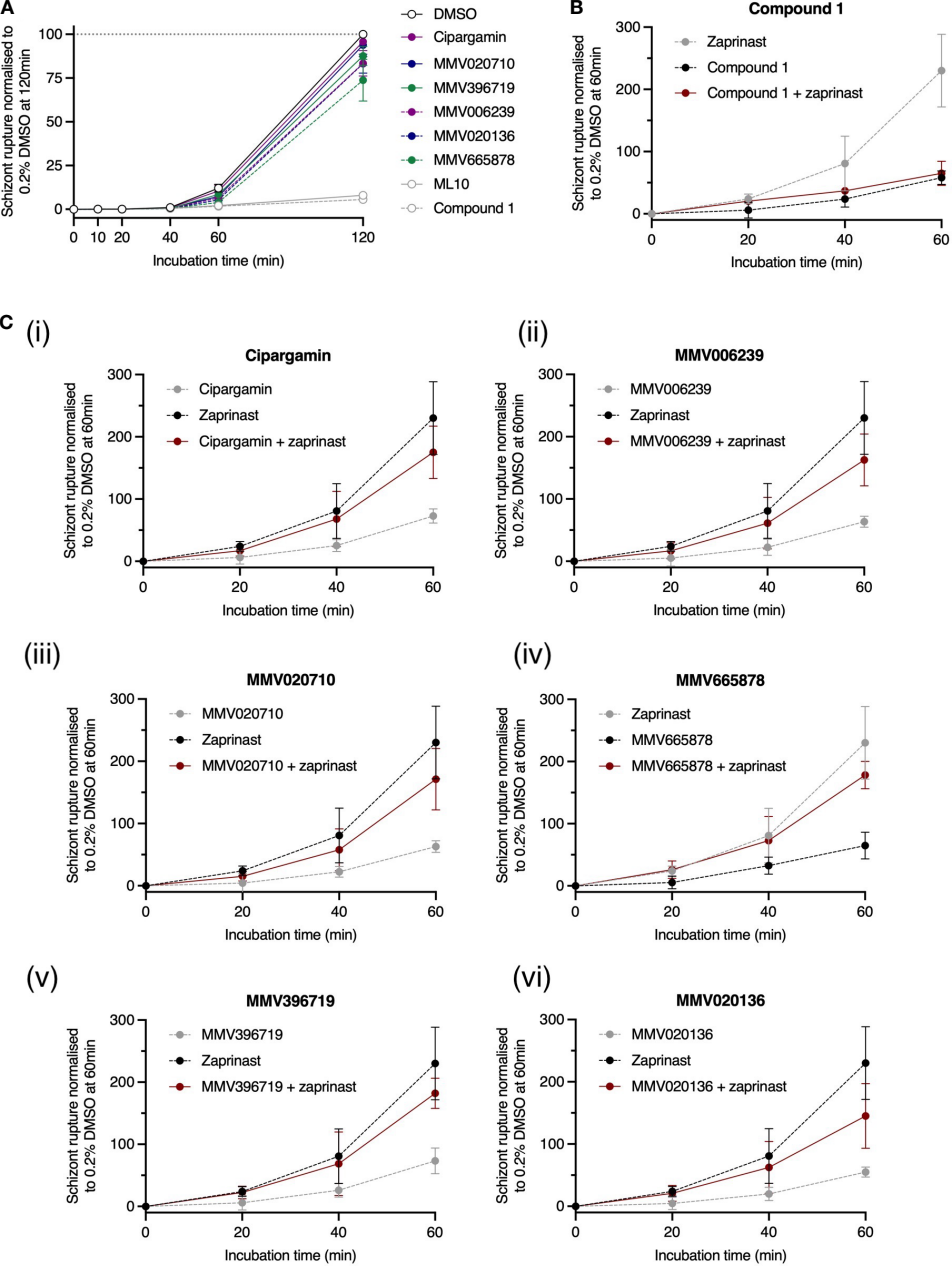
*Pf*ATP4 inhibitors likely act upstream of the cGMP/*Pf*PKG checkpoint to inhibit egress. **(A)** 3D7_Hyp1-Nluc trophozoites were grown overnight with the *Pf*PKG inhibitor ML10 (25 nM). ML10 was subsequently removed from the schizont cultures and *Pf*ATP4 inhibitors were immediately administered. Egress occurred to a similar extent in *Pf*ATP4 inhibitor-treated and vehicle control-treated cultures. As negative egress controls, ML10 (25 nM) and Compound 1 (4 μM) were (re-)administered after ML10 was first removed. **(B, C)** Premature egress and nanoluciferase release can be induced using a phosphodiesterase inhibitor, zaprinast (70 μM). The pro-egress effect of zaprinast is blocked when zaprinast is co-administered with the egress inhibitor Compound 1 **(B)**. Conversely, while egress is reduced by *Pf*ATP4 inhibitors alone (black dotted line), co-administration with zaprinast counteracts this inhibition (red solid line), resulting in enhanced egress relative to parasites treated with the DMSO vehicle control **(C)**. *Pf*ATP4 inhibitors (concentrations listed in **Figure legend 3**) and control compounds were administered to late 3D7_Hyp1-Nluc schizonts for 120 min **(A)** or 60 min **(B, C)** and RBC rupture was estimated by periodically measuring the bioluminescent signal of nanoluciferase released into the cell-free culture medium. The bioluminescent signal was normalised to that measured in DMSO-treated wells at the final timepoint after subtracting the baseline bioluminescence (measured at 0 min). Points and error bars represent the mean ± SD of three biological replicates, each consisting of technical triplicates.

Egress is preceded by a complex signal transduction pathway. One strategy for determining the point along this pathway at which a novel compound acts involves arresting the pathway at a known point using a well-characterised, reversible enzyme inhibitor. After removal of the reversible inhibitor, the novel compound is added, and we can observe whether egress is now able to proceed. If egress resumes, it is inferred that the test compound imposes a ‘barrier’ upstream of the well-characterised inhibitor. Conversely, if egress remains blocked, the novel compound must act downstream of the well-characterised inhibitor. This strategy was implemented using ML10, a potent inhibitor of *Pf*PKG (Ressurreição et al., 2020). We incubated trophozoite-stage parasites with 25 nM ML10 for ~15 h, during which time the parasites matured to the late schizont stage but were unable to egress. The ML10 was then removed and *Pf*ATP4 inhibitors, Compound 1 and ML10 were immediately added to separate aliquots of the schizont culture (**Figure 7A**). The trajectory of egress was monitored by periodically sampling the culture medium and measuring its nanoluciferase signal. In this context, nanoluciferase release (normalised to the final timepoint in DMSO-treated wells) was negligible at 10, 20 and 40 min. At 120 min, the normalised nanoluciferase signals in the presence of *Pf*ATP4 inhibitors ranged from 74 ± 12% (for MMV665878; mean ± SD) to 96 ± 0.79% (cipargamin) and were significantly higher than the nanoluciferase signals in wells treated with ML10 (8.6 ± 2.0%) and Compound 1 (6.4 ± 0.19%) (p<0.0001; one-way ANOVA with Tukey’s multiple comparisons test). These data indicate that *Pf*ATP4 inhibitors act upstream of *Pf*PKG and are not effective during the brief window between *Pf*PKG activation and merozoite release.

Egress is thought to be triggered when the intra-parasitic cGMP concentration exceeds a certain threshold required to activate cGMP-dependent *Pf*PKG, the deployment of which initiates a proteolytic signalling cascade that culminates in the rupture of the parasitophorous vacuole membrane and RBC membrane (Tan and Blackman, 2021; Dvorin and Goldberg, 2022). Guanylate cyclase α (*Pf*GCα) and phosphodiesterases, which produce and degrade cGMP respectively (Collins et al., 2013; Nofal et al., 2021), regulate the timing of this event. While normal egress likely occurs when an unknown stimulus enhances the activity of *Pf*GCα, egress can also be triggered prematurely by small-molecule phosphodiesterase inhibitors, such as zaprinast, which rapidly elevate cGMP levels, causing *Pf*PKG activation and parasite egress. In 2013, Collins et al. reported that when the *Pf*PKG inhibitor Compound 1 was co-administered with zaprinast, egress was inhibited because *Pf*PKG remained inactive despite the zaprinast-induced rise in intra-parasitic cGMP (Collins et al., 2013). Data obtained using our 3D7_Hyp1-Nluc parasites support this finding. Compound 1 in combination with zaprinast inhibited egress to an extent indistinguishable from Compound 1 alone (p≈1; one-way ANOVA with Tukey’s multiple comparisons test; **Figure 7B**). Conversely, while the *Pf*ATP4 inhibitors alone reduced egress relative to the DMSO vehicle control, co-administration of zaprinast with *Pf*ATP4 inhibitors increased egress to between 145 ± 52% (MMV020136; mean ± SD) and 182 ± 24% (MMV020719) of the vehicle control values. For five of the six *Pf*ATP4 inhibitors tested (MMV665878, MMV396719, MMV006239, MMV020710 and cipargamin), the difference between ‘zaprinast + *Pf*ATP4 inhibitor’ and ‘*Pf*ATP4 inhibitor alone’ reached significance by 60 min (p ≤ 0.026; **Figures 7C**(i-v)). MMV020136 followed a similar trend to the other MMV compounds but did not reach significance due to higher variability between replicates (**Figure 7C**(vi)). Overall, these data corroborate a model in which *Pf*ATP4 inhibitors act upstream of the cGMP checkpoint, possibly by (directly or indirectly) inhibiting the unknown stimulus that normally upregulates *Pf*GCα activity in late schizonts. By arresting the breakdown of cGMP, zaprinast sustains high cGMP levels and partially overcomes the egress blockade imposed by *Pf*ATP4 inhibitors, resulting in a level of egress between that occurring in the presence of the vehicle control and that achieved by zaprinast alone.

## 4 Discussion

In recent screens of the MMV Malaria and Pathogen Boxes, a subset of the putative *Pf*ATP4 inhibitors were found to reduce blood-stage merozoite invasion, while others inhibited egress (Lehane et al., 2014; Dennis et al., 2018b; Subramanian et al., 2018; Dans et al., 2020). This inconsistency suggested that some compounds which disrupt intraerythrocytic parasite development by inhibiting *Pf*ATP4 may dysregulate egress and/or invasion by interacting with secondary target proteins. In this study, five putative *Pf*ATP4 inhibitors from the Malaria and Pathogen Boxes were tested for inhibition of the schizont-to-ring transition, using an exported bioluminescent enzyme as a quantifiable biomarker of egress and invasion. In this study, all five compounds were found to inhibit egress, not invasion or early ring-stage development. Moreover, we demonstrated that this egress inhibition a) is dependent on extracellular Na^+^ and b) is attenuated in parasites with resistance-conferring mutations in *Pf*ATP4, thereby establishing both a mechanistic and a genetic link between egress inhibition and *Pf*ATP4 itself. Finally, we provided evidence to suggest that the *Pf*ATP4 inhibitors act upstream of the cGMP/*Pf*PKG checkpoint to block the signalling pathway that culminates in merozoite release.

To investigate the direct effects of the five MMV compounds on invasion, we administered these compounds to merozoites that had been mechanically liberated from mature schizonts. In this context, no invasion inhibition was observed, which likely reflects the fact that the time between egress and subsequent invasion (usually<1 minute *in vitro* (Weiss et al., 2015; Weiss et al., 2016)) is insufficient for Na^+^ to accumulate to toxic levels inside merozoites upon inhibition of *Pf*ATP4. After invasion, the low Na^+^ environment of the RBC likely protects merozoites and early ring-stage parasites from the Na^+^-dysregulating effects of *Pf*ATP4 inhibitors (Lee et al., 1988; Winterberg and Kirk, 2016). As the parasites transition into trophozoites and establish nutrient uptake channels in the host RBC membrane (Counihan et al., 2017; Sherling et al., 2017; Counihan et al., 2021), the entry of Na^+^ into the RBC cytoplasm through these channels renders the parasites sensitive to *Pf*ATP4 inhibitors (Gilson et al., 2019).

**Figure 8:**
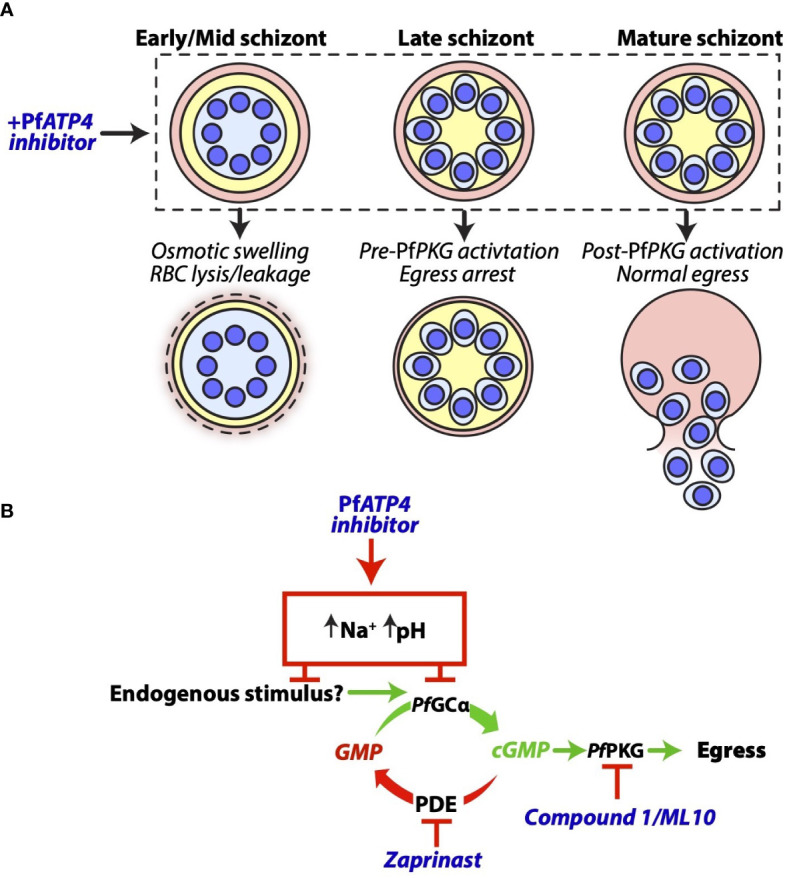
A proposed model for the effects of *Pf*ATP4 inhibitors in *P. falciparum* schizonts. **(A)** The outcomes of *Pf*ATP4 inhibition differ according to schizont age. *Pf*ATP4 inhibitors cause early- to mid-stage schizonts to accumulate Na^+^ and osmotically swell. This results in lysis or leakage of host RBCs and therefore the release of nanoluciferase into the culture medium. When administered to late schizonts, *Pf*ATP4 inhibitors arrest egress without inducing substantial lysis or leakage of host RBCs. After cGMP elevation and the deployment of *Pf*PKG, mature schizonts are no longer susceptible to *Pf*ATP4 inhibitors and egress can proceed in the presence of these compounds. **(B)** *Pf*ATP4 inhibitors act upstream of the cGMP/*Pf*PKG checkpoint to inhibit egress. During merozoite development, intra-parasitic cGMP levels are stabilised by the opposing activities of guanylyl cyclase α (*Pf*GCα) and phosphodiesterases (PDEs) An as-yet unidentified endogenous stimulus induces the functional upregulation of *Pf*GCα in late schizonts, resulting in cGMP elevation and *Pf*PKG activation. A PDE inhibitor, zaprinast, prevents cGMP degradation and therefore induces cGMP elevation and egress without the requirement for an increase in the rate of cGMP production, thereby obviating the need for the endogenous egress stimulus. Co-administration of zaprinast overcomes the egress-inhibitory effect of *Pf*ATP4 inhibitors. This supports a model wherein *Pf*ATP4 inhibitors block egress by preventing the functional upregulation of *Pf*GCα.

In contrast to free merozoites, schizonts were found to be highly susceptible to our panel of *Pf*ATP4 inhibitors. Unexpectedly, the consequences of *Pf*ATP4 inhibition were heavily dependent on schizont age and synchronicity. The *Pf*ATP4 inhibitors prevented the rupture of late-stage, segmented schizonts in a concentration-dependent manner. However, when administered to early- to mid-stage schizonts or relatively asynchronous schizont cultures, increasing compound concentrations promoted cell rupture and nanoluciferase release (**Figure 8A**). Replacement of extracellular Na^+^ with K^+^ and sucrose partially protected late-stage schizonts from egress inhibition, and partially protected early- to mid-stage schizonts from compound-induced rupture. Multiple *Pf*ATP4 inhibitors have been shown to cause swelling (Jiménez-Díaz et al., 2014; Dennis et al., 2018a; Dennis et al., 2018b) and lysis (Jiménez-Díaz et al., 2014; Gilson et al., 2019) of parasites and parasitised RBCs, likely due to water uptake by osmosis. Likewise, the five MMV compounds studied here caused trophozoite-infected RBCs to release nanoluciferase in the presence of extracellular Na^+^, presumably due to swelling of the trophozoites and subsequent lysis (or leakage) of the host cell. Given that lysis of parasitised RBCs acts as a confounding factor when measuring extracellular nanoluciferase as a biomarker of egress, the failure to classify several putative *Pf*ATP4 inhibitors as egress inhibitors in the aforementioned nanoluciferase-based screen of the Pathogen Box (Dans et al., 2020) is likely attributable to host cell lysis. We have demonstrated that *Pf*ATP4 inhibition causes both Na^+^-dependent lysis and Na^+^-dependent egress inhibition and that the net effect of these opposing factors on nanoluciferase release depends on schizont age. This led to wide assay-to-assay variability in the observable effects of *Pf*ATP4 inhibition when the treated schizont populations had not been tightly synchronised. Consistent with this observation, the mean standard deviation (across biological triplicates) for egress inhibition by the putative *Pf*ATP4 inhibitors (Dennis et al., 2018b) in the Pathogen Box screen was 24%, which was substantially higher than that of the remaining compounds (11%) (Dans et al., 2020).

The timing of egress is dictated by the balance between cGMP production by *Pf*GCα and degradation by phosphodiesterases (Yuasa et al., 2005; Howard et al., 2015; Baker et al., 2017; Nofal et al., 2021). Egress is thought to occur when an as-yet unidentified endogenous stimulus upregulates the activity of *Pf*GCα, resulting in cGMP elevation and activation of cGMP-dependent *Pf*PKG (Gurnett et al., 2002; Tan and Blackman, 2021; Dvorin and Goldberg, 2022). An ensuing proteolytic cascade culminates in sequential rupture of the parasitophorous vacuole and RBC membranes (Hale et al., 2017; Thomas et al., 2018). Zaprinast, by inhibiting phosphodiesterases (Howard et al., 2015), obviates the need for the endogenous egress stimulus, causing premature cGMP elevation and downstream egress (**Figure 8B**). Co-administration of zaprinast with a compound that blocks the endogenous stimulus would therefore be expected to still permit egress. As such, our finding that zaprinast counteracted the egress inhibition imposed by *Pf*ATP4 inhibitors suggested that *Pf*ATP4 inhibition disrupts this unknown endogenous stimulus, thereby preventing the functional upregulation of *Pf*GCα in late schizonts. Consistent with this hypothesis, schizonts which had been stalled using a *Pf*PKG inhibitor, ML10 (Ressurreição et al., 2020), were able to resume egress when ML10 was removed and replaced by *Pf*ATP4 inhibitors, indicating that the *Pf*ATP4 inhibitors act upstream of *Pf*PKG. In 2014, Vaidya et al. reported that mutations in both *Pf*ATP4 and Ca^2+^-dependent protein kinase 5 (*Pf*CDPK5) were required for full resistance to the pyrazoleamide antimalarial PA21A050 (Vaidya et al., 2014). *Pf*CDPK5 likely cooperates with *Pf*PKG to mediate egress (Dvorin et al., 2010; Absalon et al., 2018) and it is plausible that mutations in *Pf*CDPK5 facilitate egress despite *Pf*ATP4 inhibitor-induced suppression of the *Pf*GCα/cGMP/*Pf*PKG pathway.

Recent insights into the function of GCα in Apicomplexan parasites offer potential clues as to the mechanism by which *Pf*ATP4 inhibition prevents egress from late schizonts. *Pf*GCα consists of a GC domain appended to a P4-ATPase-like domain, an atypical structure that is conserved among Ciliophora and Apicomplexans (Linder et al., 1999; Nofal et al., 2021). In *P. falciparum* and the closely related Apicomplexan parasite *Toxoplasma gondii* (*T. gondii*), the P4-ATPase domain is essential and likely regulates cGMP production by the GC domain (Yang et al., 2017; Bisio et al., 2019; Nofal et al., 2021). In other organisms, P4-ATPases predominantly function as flippases, translocating phospholipids within membranes to generate bilayer asymmetry which contributes to intracellular signal transduction (Takada et al., 2018). Interestingly, two *Pf*ATP4 inhibitors (cipargamin and PA21A050) have been shown to rapidly induce cholesterol accumulation and aberrant clustering of glycosylphosphatidylinositol-anchored proteins in *P. falciparum* membranes (Das et al., 2016). Moreover, the majority of the putative *Pf*ATP4 inhibitors in the Malaria and Pathogen Boxes, including those utilised in this study, have been found to sensitise intra-erythrocytic *P. falciparum* to the cholesterol-dependent detergent saponin, suggesting that these compounds also cause cholesterol to accumulate in the parasite plasma membrane (Bhatnagar et al., 2019). Given that Apicomplexan GCs appear to respond to lipid-based signals (Bisio et al., 2019; Paul et al., 2020), it is conceivable that the lipid perturbations induced by *Pf*ATP4 inhibitors dysregulate or counteract the normal lipid translocation activity of the P4-ATPase domain, thereby hindering its capacity to activate the GC domain.

Many structurally diverse antimalarials act by inhibiting *Pf*ATP4 (Spillman and Kirk, 2015), including two compounds currently in clinical trials (National Library of Medicine (U. S.), 2022b; National Library of Medicine (U. S.), 2022a). There is an incentive to describe the precise MoA of these compounds as this will aid in pre-empting resistance mechanisms and devising appropriate combination therapies. Whereas earlier work primarily focussed on the effects of *Pf*ATP4 inhibition on intra-erythrocytic parasite development (Vaidya et al., 2014; Das et al., 2016; Dennis et al., 2018a; Gilson et al., 2019), this study has confirmed that *Pf*ATP4 activity is also required for egress. Further work to identify the proteins and processes that regulate or depend on Na^+^ homeostasis in *P. falciparum* could inform the development of novel antimalarials that will synergise with *Pf*ATP4 inhibitors to potently arrest merozoite egress.

## Data availability statement

The original contributions presented in the study are included in the article/**Supplementary Material**. Further inquiries can be directed to the corresponding author.

## Author contributions

CB, MD, TJ, and PG conceived this research and designed the experiments. CB conducted the experiments and generated the figures. PG prepared **Figure 8**. CB wrote the manuscript. PG, MD, and TJ edited the manuscript. BC and PG provided funding. All authors have approved the submitted manuscript.

## Funding

This work was supported by funding from the Victorian Operational Infrastructure Support Program received by the Burnet Institute. Funding was provided by the National Health and Medical Research Council (grant numbers APP2001073, APP1185354 and 119780521). MD is a recipient of an Australian Government Research Training Scholarship and TJ is a recipient of the Melbourne Research Scholarship.

## Acknowledgments

The authors would like to thank the Medicines for Malaria Venture (MMV) for providing access to the MMV Malaria and Pathogen Boxes. We gratefully acknowledge the Melbourne branch of the Australian Red Cross Blood Bank for providing human blood. We thank Dr. Coralie Boulet for contributing to the conception and design of experiments and Molly P. Schneider for technical assistance.

## Conflict of interest

The authors declare that the research was conducted in the absence of any commercial or financial relationships that could be construed as a potential conflict of interest.

## Publisher’s note

All claims expressed in this article are solely those of the authors and do not necessarily represent those of their affiliated organizations, or those of the publisher, the editors and the reviewers. Any product that may be evaluated in this article, or claim that may be made by its manufacturer, is not guaranteed or endorsed by the publisher.
