# Supplementary material for: PfATP4 inhibitors in the Medicines for Malaria Venture Malaria Box and Pathogen Box block the schizont-to-ring transition by inhibiting egress rather than invasion
Source: Front Cell Infect Microbiol. 2022 Nov 30;12:1060202. doi: 10.3389/fcimb.2022.1060202 (PMC9747762; doi:10.3389/fcimb.2022.1060202)
Supplement: Supplementary file 2 [file Presentation_1.pdf]

frontiers

Supplementary Figures

MMV665878 (Malaria Box)<br>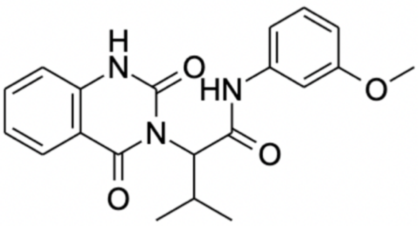	MMV396719 (Malaria Box)<br>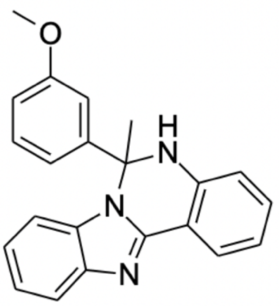
MMV006239 (Pathogen Box)<br>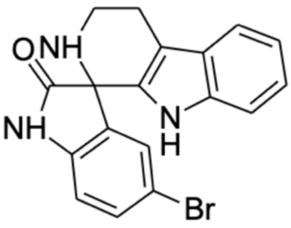	MMV020136 (Pathogen Box)<br>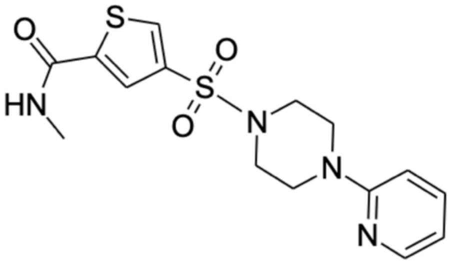
MMV020710 (Pathogen Box)<br>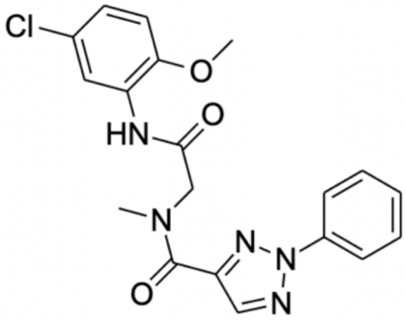	Cipargamin<br>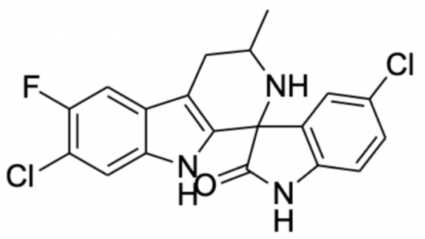
MB14<br>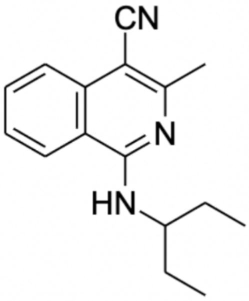	

Supplementary Figure 1. Chemical structures of the putative *Pf*ATP4 inhibitors used in this study. Cipargamin was formerly known as NITD609 or KAE609. MB14 was formerly known as Compound 25[85]. Chemical structures were prepared using ChemDraw.

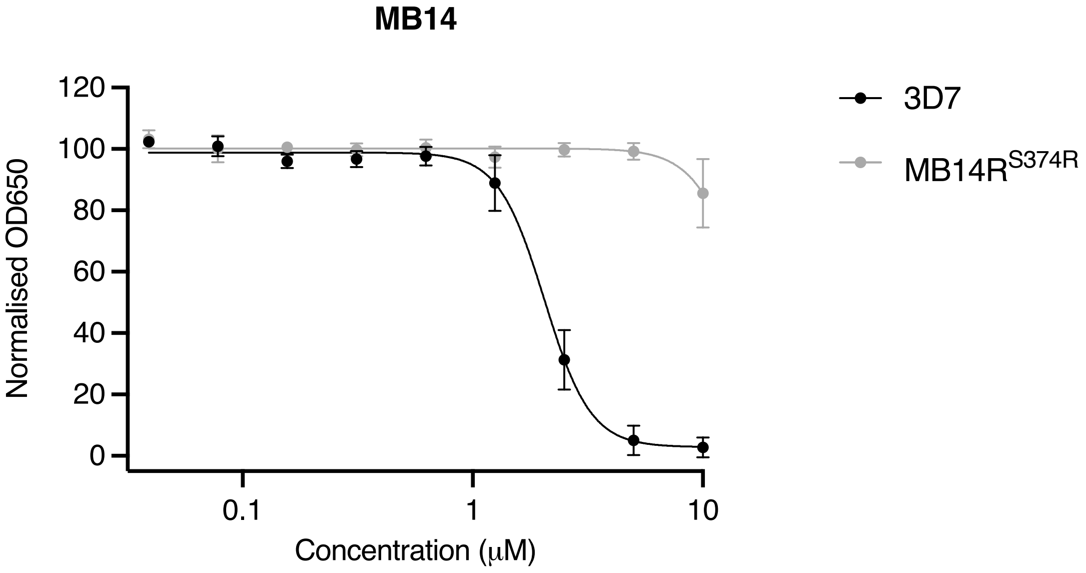

Supplementary Figure 2. A point mutation in *Pf*ATP4 confers resistance to MB14. 3D7 and MB14R^{S374R} parasites were exposed to a 2-fold dilution series of MB14 for 72 h. Lactate dehydrogenase activity was then measured as a biomarker of parasite proliferation. The optical densities at 650 nm (OD650) of drug-treated samples were normalised to the OD650 of vehicle control-treated samples after subtracting the OD650 of a background control (uninfected RBCs). Points and error bars represent the mean ± SD of three biological replicates, each consisting of technical triplicates.

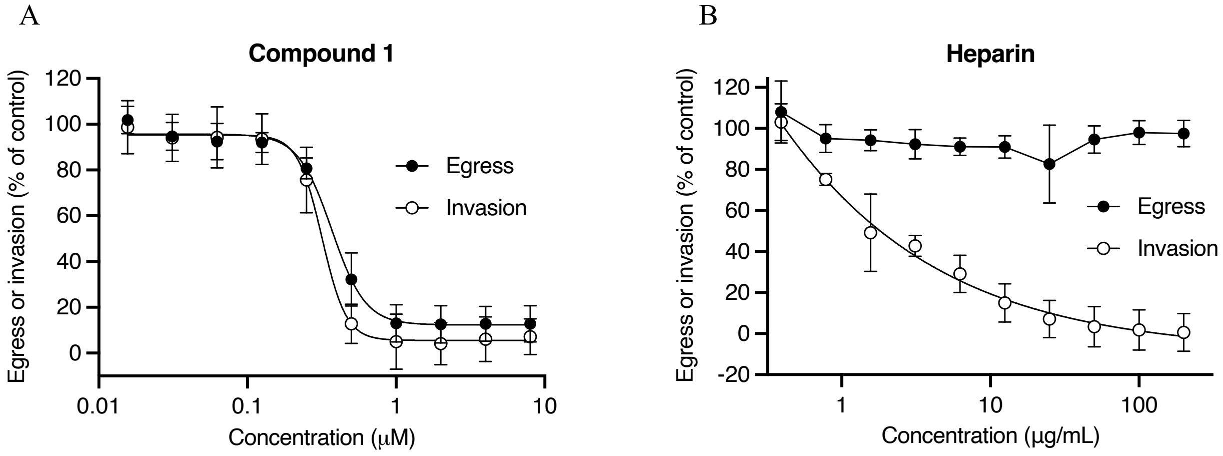

Supplementary Figure 3. The control compounds Compound 1 and heparin inhibit egress and invasion, respectively, in a concentration-dependent manner. In the "nanoluciferase egress and invasion assay", 3D7_Hyp1-Nluc schizonts were exposed to a 2-fold dilution series of the egress inhibitor Compound 1 **(A)** or the invasion inhibitor heparin **(B)**. Egress was quantified after 4 h by measuring the bioluminescent signal intensity of nanoluciferase released into the extracellular medium. Drugs were then removed and unruptured schizonts were lysed by sorbitol treatment. Invasion was quantified 24 h later by chemically lysing the resultant trophozoite cultures and measuring the total nanoluciferase signal of the lysate. Points and error bars represent the mean ± SD of three biological replicates, each consisting of technical duplicates.

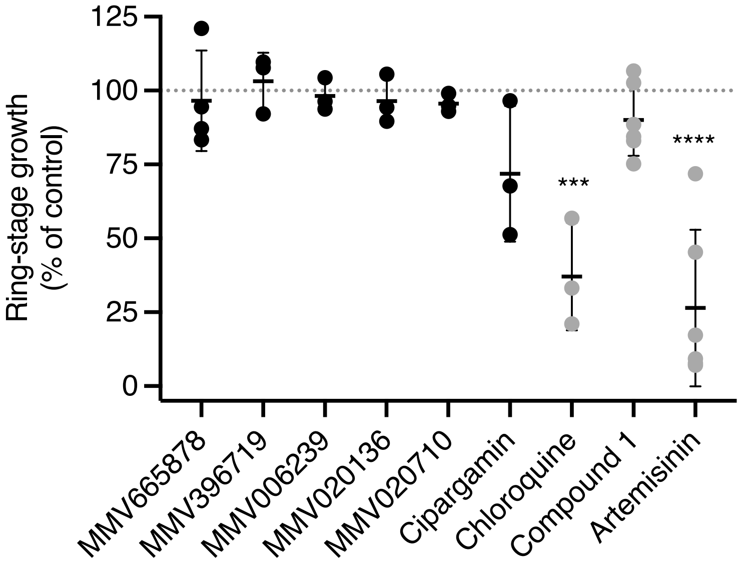

Supplementary Figure 4. *Pf*ATP4 inhibitors do not irreversibly inhibit early ring-stage parasite development. Early ring-stage 3D7_Hyp1-Nluc parasites (~0-4 hpi) were treated with *Pf*ATP4 inhibitors at ~10×IC_{50} (0.7 µM MMV665878, 3.2 µM MMV396719, 2 µM MMV006239, 3 µM MMV020136, 0.6 µM MMV020710, 10 nM cipargamin) for 4 h. After removal of compounds, parasites were incubated for 24 h and the parasites' capacity to mature to the trophozoite stage was ascertained by measuring the total nanoluciferase signals of culture lysates. *Pf*ATP4 inhibitors did not significantly inhibit parasite development relative to the negative control drug, Compound 1 (4 µM; ~10×IC_{50}). The known ring-stage development inhibitors chloroquine (75 nM; ~5×IC_{50}) and artemisinin (25 nM; ~8×IC_{50}) caused a significant reduction in nanoluciferase signal. Points and error bars represent the mean ± SD of at least three biological replicates, each consisting of technical triplicates. Statistical analysis was performed via one-way ANOVA with Dunnett's multiple comparisons test for comparison to the negative control, Compound 1. ***$p<0.001$, ****$p<0.0001$. No asterisk indicates not significant.

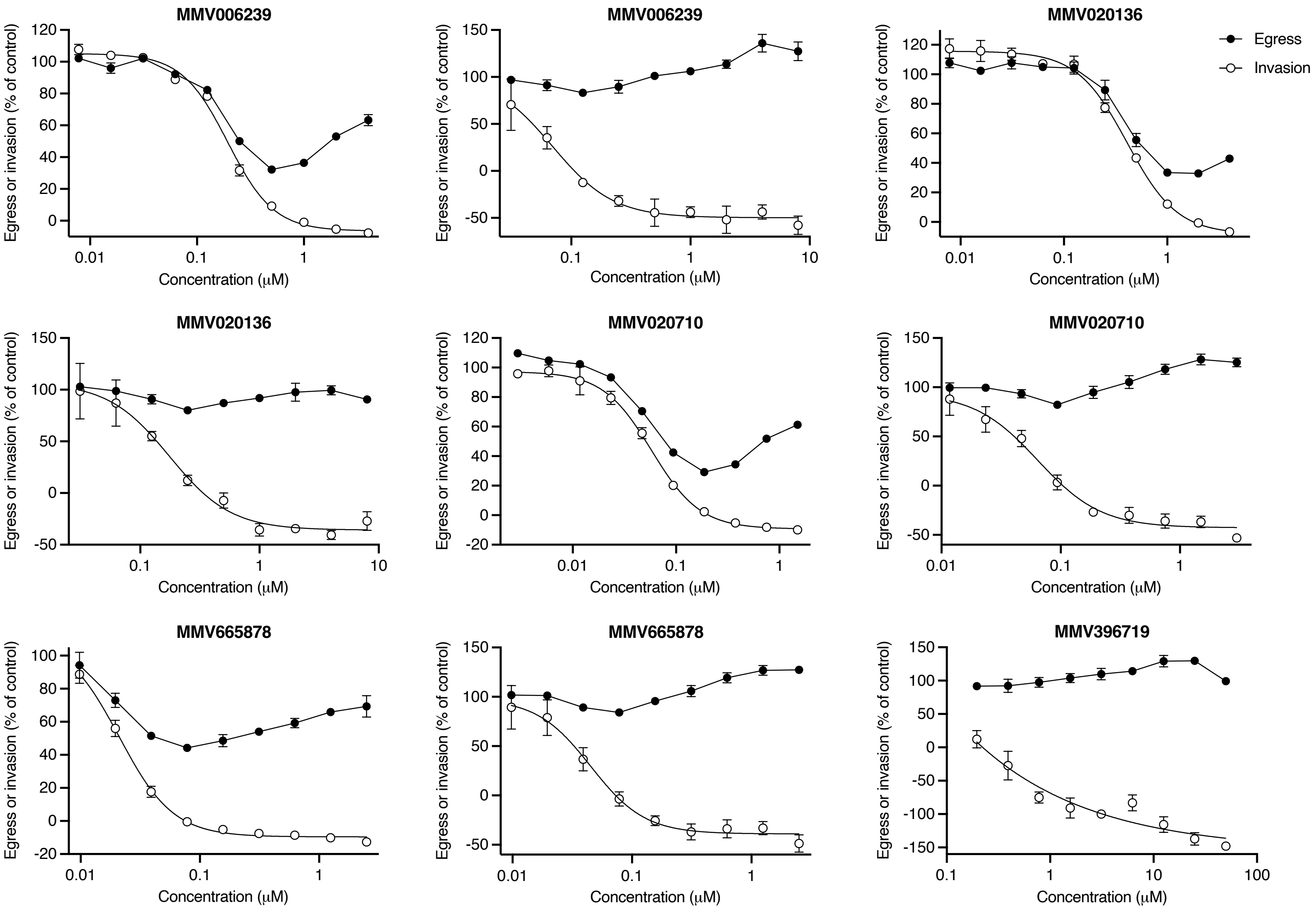

Supplementary Figure 5. When administered to schizonts with a wide age range (~35-42hpi), high concentrations of *Pf*ATP4 inhibitors do not inhibit nanoluciferase release but do reduce subsequent invasion. The "nanoluciferase egress and invasion assay" was conducted using sorbitol-synchronised 3D7_Hyp1-Nluc schizonts. Each graph shows data obtained in a single experiment. Points and error bars represent the mean ± SD of technical duplicates or triplicates. See also Figure 2, left-hand column,

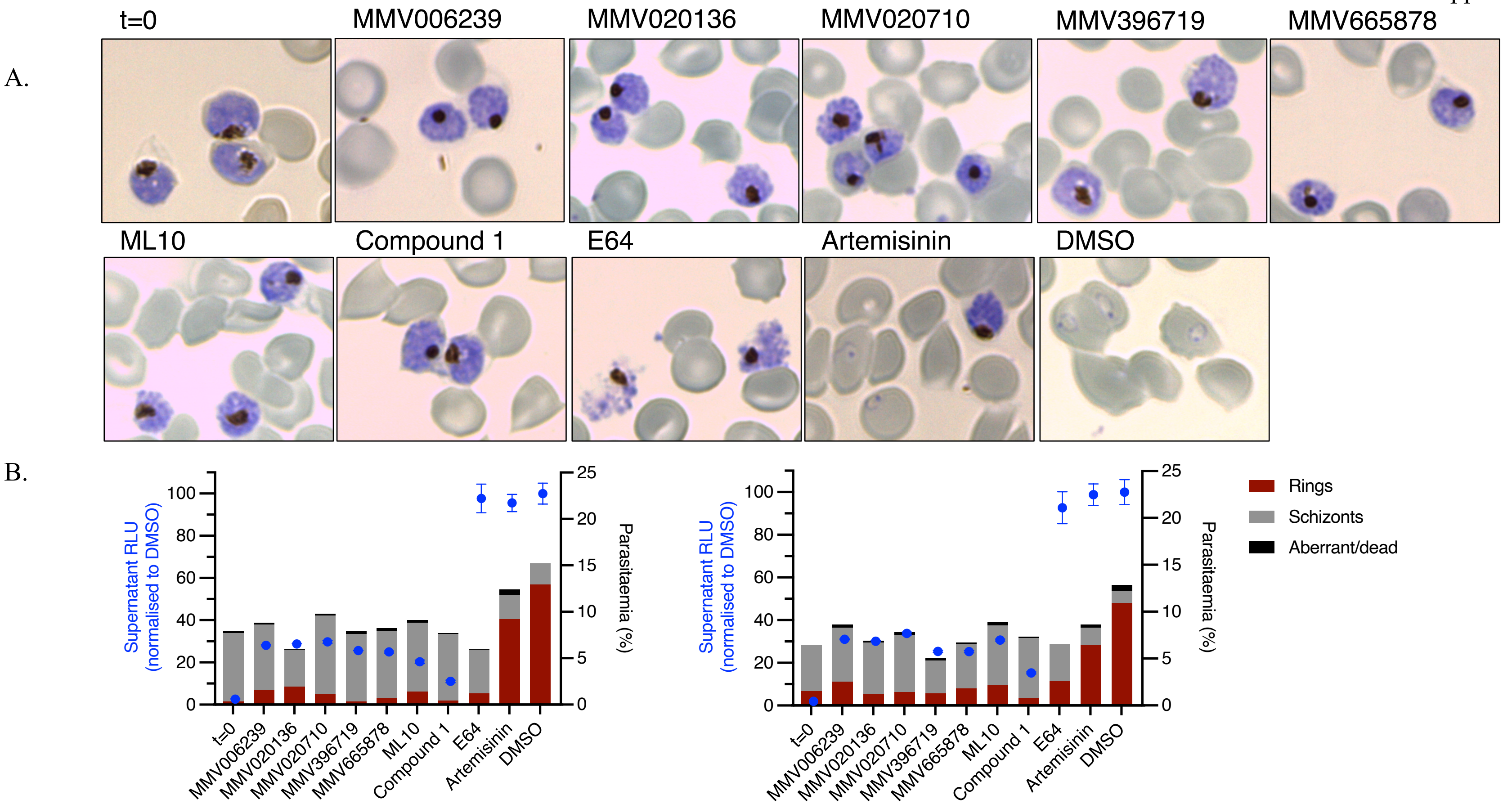

Supplementary Figure 6. When administered to synchronous late-stage schizonts, *Pf*ATP4 inhibitors reduce RBC rupture and merozoite egress. Synchronous late-stage schizonts were treated with ~10×IC_{50} of *Pf*ATP4 inhibitors (0.7 µM MMV665878, 3.2 µM MMV396719, 2 µM MMV006239, 3 µM MMV020136, 0.6 µM MMV020710), ML10 (25 nM), Compound 1 (4 µM), E64 (10 µM) or artemisinin (30 nM) for 4 h in triplicate wells of 96-well U-bottom plates.. **(A)** Representative Giemsa images of parasite cultures after 4 h treatment show that the *Pf*ATP4 inhibitors produce an egress inhibition phenotype comparable to ML10 and Compound 1. **(B)** Following treatment, cell-free supernatant was harvested and the bioluminescent signal intensity of nanoluciferase released into the supernatant was measured in relative light units (RLU). Points and error bars represent the mean ± SD of technical triplicates. Giemsa-stained thin blood smears were prepared from duplicate wells and the parasitaemia was estimated by counting 1000 cells. E64 inhibited merozoite egress but permitted RBC membrane perforation and leakage of nanoluciferase. Artemisinin, a potent antimalarial which does not specifically target egress, moderately reduced new ring formation relative to the vehicle control (0.04% DMSO) but did not reduce nanoluciferase release. Each graph shows data generated in a single independent experiment.

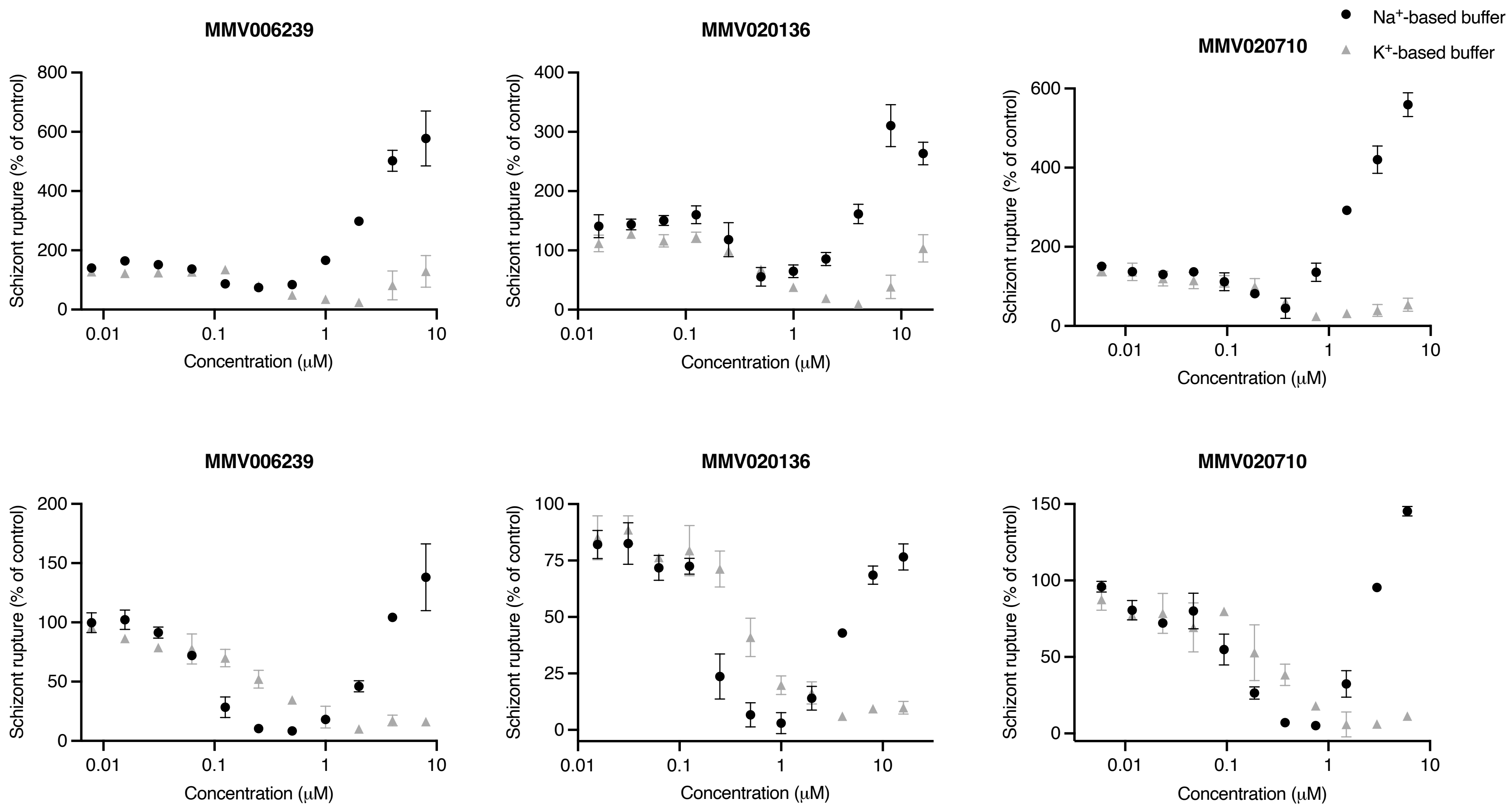

Supplementary Figure 7. When administered to early/mid-stage schizonts, high concentrations of *Pf*ATP4 inhibitors promote nanoluciferase release in a Na^{+}-dependent manner. Prior to assays, 3D7_Hyp1-Nluc parasites were synchronised to a 2 h age range and grown to ~38-40 hpi. Schizonts were resuspended in Na^{+}-based buffer (black circles) or a K^{+}-based buffer (grey triangles) and exposed to a 2-fold dilution series of compounds of interest for 4 h. Cell-free supernatant was then harvested and the extracellular nanoluciferase activity was measured. Values were normalised to the extracellular nanoluciferase activity of samples treated with the DMSO vehicle control. Each graph shows data obtained in a single experiment. Points and error bars represent the mean ± SD of technical duplicates. See also Figure 5.

A

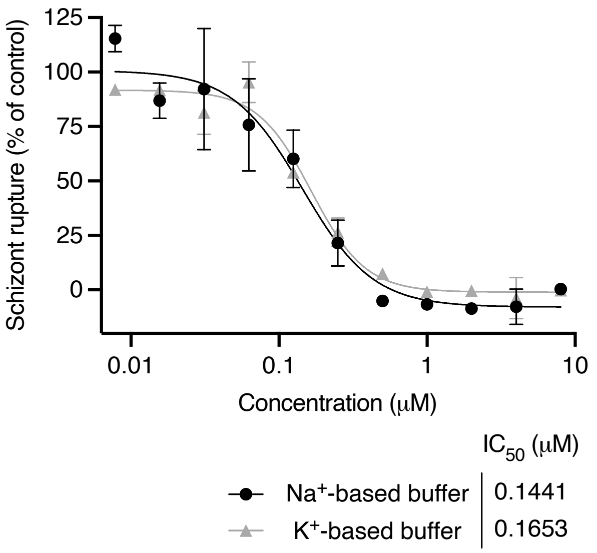

B

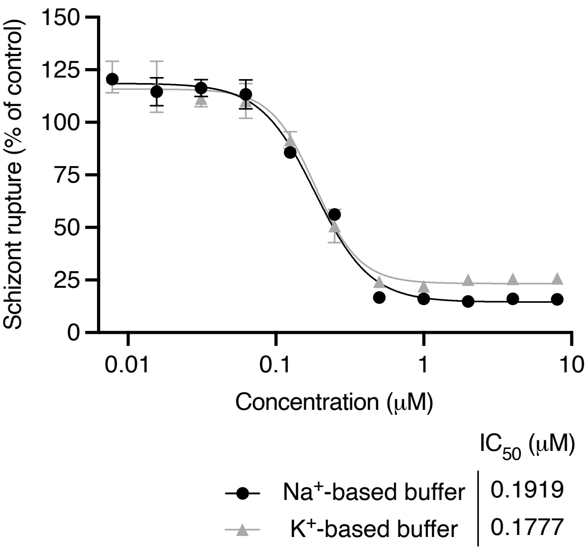

C

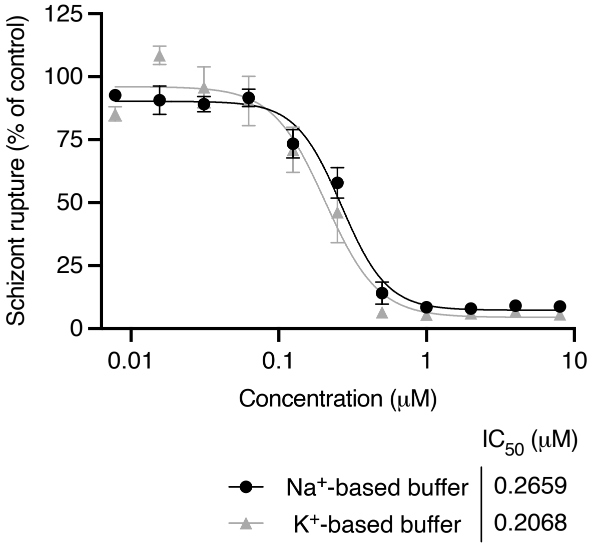

Supplementary Figure 8. Compound 1 behaves as a concentration-dependent egress inhibitor in Na^+-based and K^+-based buffer regardless of schizont age. Prior to assays, 3D7_Hyp1-Nluc parasites were synchronised to a 2 h age range and grown to ~36-40 hpi **(A-B)** or 40-42 hpi **(C)**. Schizonts were resuspended in Na^+-based buffer (black circles) or a K^+-based buffer (grey triangles) and exposed to a 2-fold dilution series of compounds of interest for 4 h. Cell-free supernatant was then harvested and the extracellular nanoluciferase activity was measured. Values were normalised to the extracellular nanoluciferase activity of samples treated with the DMSO vehicle control. Each graph shows data obtained in a single experiment. Points and error bars represent the mean ± SD of technical duplicates.
